# Alternative Translation Initiation in *PRKN* Delays the Onset of Parkinson's Disease and Offers a Therapeutic Target

**DOI:** 10.1002/ana.78180

**Published:** 2026-02-22

**Authors:** Arian Hach, Katja Lohmann, Manabu Funayama, Poornima J. Menon, Eva‐Juliane Vollstedt, Teresa Kleinz, Hiroyo Yoshino, Suzanne Lesage, Britta Meier, Carolyn M. Sue, Jean‐Christophe Corvol, Alexis Brice, Nobutaka Hattori, Christine Klein, Aleksandar Rakovic

**Affiliations:** ^1^ Institute of Neurogenetics University of Lübeck Lübeck Germany; ^2^ Research Institute for Diseases of Old Age, Graduate School of Medicine Juntendo University Tokyo Japan; ^3^ Department of Neurology Faculty of Medicine, Juntendo University Tokyo Japan; ^4^ Sorbonne Université, Institut du Cerveau – Paris Brain Institute – ICM, Inserm, CNRS Department of Neurology, Pitié‐Salpêtrière Hospital Paris France; ^5^ Faculty of Medicine University of New South Wales Sydney Australia; ^6^ Neuroscience Research Australia Sydney New South Wales Australia; ^7^ Neurodegenerative Disorders Collaborative Laboratory RIKEN Center for Brain Science Saitama Japan

## Abstract

**Objective:**

Biallelic variants in *PRKN* cause autosomal recessive Parkinson's disease (PD) with a median age at onset of 31 years. When evaluating the 16 previously published carriers of a homozygous deletion of Exon 2 from the International Parkinson's Disease and Movement Disorder Society Gene Database (MDSGene) database, the median age at onset is later (39.5 years) than in carriers of other *PRKN* pathogenic variants. We investigated whether these carriers show delayed disease onset compared with carriers of other pathogenic *PRKN* variants and explored the underlying molecular mechanism.

**Methods:**

We compared 26 homozygous *PRKN* Exon 2 deletion carriers with carriers of other pathogenic variants. Using human‐induced pluripotent stem cell (hiPSC)‐derived neuronal cell models from an unaffected 86‐year‐old carrier, genome‐edited control lines, neuroblastoma cell lines, and in silico prediction, we investigated the underlying mechanism.

**Results:**

Patients with *PRKN* Exon 2 deletions showed a later age at onset compared with carriers of other pathogenic variants. We discovered elevated levels of an N‐terminally truncated Parkin proteoform lacking amino acids 1–79 due to internal translation initiation. This truncated protein partially retained ubiquitin ligase activity at endogenous levels. Treatment with Parkin modulator BIO‐2007817 enhanced this residual function but reduced endogenous full‐length Parkin activity.

**Interpretation:**

Residual truncated Parkin function provides a molecular explanation for a delayed disease onset in *PRKN* Exon 2 deletion carriers. Whereas this retained activity can be pharmacologically enhanced, the modulator's inhibitory effect on endogenous full‐length Parkin may mandate strict patient stratification based on genotype. This finding offers mutation‐specific counseling opportunities and highlights a potential therapeutic approach for appropriately selected patients with PARK‐*PRKN*. ANN NEUROL 2026;99:1379–1393

Whereas the majority of patients with Parkinson's disease (PD) are classified as idiopathic with an unknown origin, monogenic causes constitute up to 15% of patients.^1^, ^2^ Of these, pathogenic variants in the *PRKN* gene are the most common cause of familial, monogenic autosomal recessive PD (PARK‐*PRKN*).^1^, ^2^

PD is typically characterized by a relatively late median age at disease onset (AAO) of 60 years, with increasing prevalence at higher ages.^3^ Patients with a monogenic cause of PD usually develop the disease at an earlier AAO. For instance, the median AAO for PARK‐*PRKN* is 31 years, notably, with a range from 3 to 81 years.^4^, ^5^, ^6^ Although no variant‐specific genotype–phenotype correlation has been detected yet, there is a 3 to 5 year earlier AAO in carriers of frameshift or structural variants compared with carriers of missense variants in *PRKN*.^7^ Furthermore, N‐terminal variants are associated with an overall earlier AAO.^7^

*PRKN* encodes the Parkin RBR E3 ubiquitin protein ligase. This E3 ubiquitin ligase is recruited to the outer mitochondrial membrane^8^ and activated by phosphorylation of its regulatory Ubiquitin‐like (Ubl) domain via *PINK1* upon damage‐induced mitochondrial membrane depolarization.^9^, ^10^, ^11^, ^12^ The disease mechanism is considered to be a loss of function.^13^, ^14^, ^15^ Interestingly, Parkin constructs lacking the entire Ubl domain exhibit residual ubiquitination activity.^16,17^ Furthermore, a truncated form of Parkin missing its Ubl domain (Parkin^Δ1–79^) is translated from an internal translation initiation site (TIS) in Exon 3 at position p.Met80 (NM_004562.3/NP_004553.2; Fig 1).^18^, ^19^, ^20^ This form is stably detectable, albeit at a low level, in human brain homogenates and cultured cells with or without *PRKN* variants.^19^, ^20^

**FIGURE 1:**
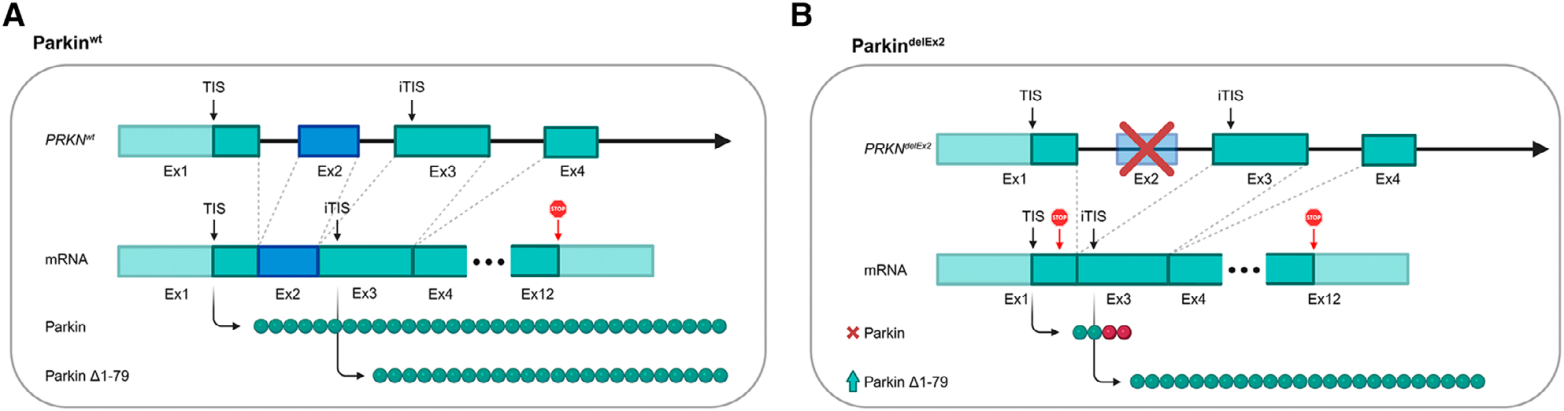
Parkin^Δ1–79^ molecular mechanism. (A) The *PRKN* ^wt^ allele transcribes to an mRNA transcript harboring both the canonical TIS and the iTIS in Exon 3. Thus, in addition to the predominantly expressed canonical full‐length Parkin, carriers of wild‐type alleles also express the N‐terminally truncated Parkin^Δ1–79^ variant, albeit to a lesser degree. (B) The *PRKN* ^delEx2^ allele results in a shortened and frameshifted mRNA transcript in which a premature stop codon closely follows the canonical TIS. This likely represents an unfavorable nucleotide context for translation initiation and may shift translation toward the iTIS. We hypothesize that a TIS favorability shift and closer proximity of the internal translation initiation site to the 5′ cap facilitates increased levels of Parkin^Δ1–79^ in biallelic *PRKN* ^delEx2^ carriers. The figure was created using BioRender. iTIS = internal translation initiation site; TIS = translation initiation site. [Color figure can be viewed at www.annalsofneurology.org]

We hypothesized that carriers of pathogenic *PRKN* variants upstream of the internal TIS benefit from residual Parkin^Δ1–79^ activity, leading to a milder phenotype and later AAO. In this study, we indeed demonstrate that carriers of a homozygous Exon 2 deletion in *PRKN* (*PRKN* ^delEx2^) have a later median AAO compared with other patients with PARK‐*PRKN*. Furthermore, we show elevated Parkin^Δ1–79^ levels in cells lacking Exon 2 due to the closer proximity of the internal TIS to the conventional start of translation of *PRKN*. Using induced dopaminergic midbrain neurons derived from a previously reported asymptomatic homozygous *PRKN* ^delEx2^ carrier^21^ and CRISPR/Cas9‐edited homozygous *PRKN* ^delEx2^ isogenic lines, we showed remaining E3 ubiquitin ligase activity which was absent in the carriers of pathogenic variants downstream of the internal TIS. Importantly, a recently characterized allosteric Parkin modulator^22^, ^23^ increased Parkin^Δ1–79^ activity in our models. At the same time, it reduced endogenous full‐length Parkin activity, highlighting the need for careful dose optimization and patient stratification in future variant‐specific therapeutic strategies.

## Methods

### Sample and Cohort Description

To investigate the effect of a biallelic *PRKN* ^delEx2^ pathogenic variant on AAO, PARK*‐PRKN* patient data from multiple sources were aggregated. Information on biallelic *PRKN* variant carriers curated in the International Parkinson's Disease and Movement Disorder Society Gene Database (MDSGene) served as the primary data source.^6^ In addition, a recently published PD cohort^24^ and a case report^25^ describing biallelic *PRKN* ^delEx2^ variant carriers were included. Furthermore, comprehensive individual data on additional homozygous *PRKN* ^delEx2^ variant carriers were collected across multiple research centers through a collaboration with the Department of Neurology, Faculty of Medicine, Juntendo University,^26^ the French Parkinson's Disease Genetics Study Group (PDG),^7^ and the MJFF Global Genetic Parkinson's Disease Project.^27^ We collected demographic and clinical data for 512 patients with PARK‐*PRKN*, including 26 with a homozygous *PRKN* ^delEx2^ variant and 485 with other variants. One of these patients had not been previously reported (Table 1). All 26 patients with biallelic *PRKN* ^delEx2^ were matched to other variant carriers on propensity score (Supplementary Table S1; see statistics chapter in Supplementary Data S1).

**TABLE 1:** Demographics of Patients With a Homozygous *PRKN* ^delEx2^ Variant for *PRKN* (NM_004562.3) Included in the AAO Analysis

ID	Country	Sex	AAO	AAE	Previously Reported	Previously Included in MDSGene (April 2025)	Match Subclass
190	Turkey	M	31	38	Yes^7^	No	17
792	Japan	M	21	54	No	No	7
957	Japan	M	42	45	Yes^26^	No	8
1,490	Japan	F	46	71	Yes^26^	No	9
1,610	Japan	M	42	53	Yes^26^	No	10
1,611	Japan	M	48	60	Yes^26^	No	11
1,655	Japan	M	38	48	Yes^26^	No	12
3,248	Japan	F	35	54	Yes^26^	No	13
4,224	Japan	F	53	66	Yes^26^	No	14
4,225	Japan	M	47	63	Yes^26^	No	15
4,662	Japan	F	32	54	Yes^26^	No	16
761,372,890	Turkey	M	41	NA	Yes^7^	No	25
VIE‐PD3322	Turkmenistan	F	14	NA	Yes^7^	No	26
AR‐102	China	M	19	NA	Yes^24^	No	18
AR‐168	China	F	29	NA	Yes^24^	No	19
AR‐180	China	F	24	NA	Yes^24^	No	20
EOPD‐0408	China	M	13	NA	Yes^24^	No	21
EOPD‐0615	China	M	17	NA	Yes^24^	No	22
EOPD‐0633	China	F	29	NA	Yes^24^	No	23
D81	Japan	M	60	81	Yes^25^	No	24
5	Spain	M	37	51	Yes^42^	Yes	2
4	Japan	M	50	51	Yes^43^	Yes	5
7	Spain	M	44	51	Yes^44^	Yes	1
Patient 2	China	M	36	40	Yes^45^	Yes	3
8	Japan	M	45	70	Yes^46^	Yes	6
Cas232	Spain	M	51	NA	Yes^47^	Yes	4

AAO = age at onset; AAE = age at examination; MDSGene = Movement Disorder Society Gene Database; NA = not available (not reported).

Using human dermal fibroblasts of one homozygous *PRKN* ^delEx2^ carrier, 2 healthy donors, and 4 carriers of other homozygous or compound heterozygous *PRKN* variants, we assessed endogenous Parkin activity in vitro (Supplementary Table S2). Additionally, human‐induced pluripotent stem cells (hiPSC)‐derived midbrain dopaminergic neurons (iDNs) and small molecule neuronal precursor cells (smNPCs) originating from one homozygous *PRKN* ^delEx2^ carrier, 2 healthy controls, 1 carrier of a homozygous *PRKN* Exon 7 deletion, as well as genome‐edited lines derived from the controls carrying homozygous Exon 2 or Exon 3 deletions were analyzed (Supplementary Table S3).

### Cell Culture

SH‐SY5Y, HEK 293FT, and primary human dermal fibroblasts were cultured in Dulbecco's Modified Eagle Medium containing 10% fetal bovine serum and 1% penicillin/streptomycin (all from Gibco). Cells were passaged when they reached 80 to 90% confluence. Fibroblast passage numbers were < 15 and varied by ≤2.

To disrupt the mitochondrial membrane potential, SH‐SY5Y cells, human dermal fibroblasts, and dopaminergic midbrain neurons were treated with 1 μM valinomycin (Sigma‐Aldrich) for 3, 6, or 14 hours, respectively. For proteasome inhibition, SH‐SY5Y overexpression models were preincubated with 10 μM MG132 (Sigma‐Aldrich) for 30 minutes. Viral transduction details are found in Supplementary Data S1.

The hiPSCs were maintained on Matrigel‐coated plates in mTeSR Plus (STEMCELL Technologies) and passaged manually at 70 to 80% confluence. Generation, characterization, and genome editing of hiPSCs are described in Supplementary Data S1. Differentiation of characterized hiPSCs (Supplementary Fig S1) into midbrain dopamine neurons and small molecule neuronal precursor cells (smNPCs) was performed following established protocols with minor alterations.^28^, ^29^ Differentiation paradigm details are provided in Supplementary Data S1.

### Western Blotting

For Western blots, proteins were isolated using RIPA buffer supplemented with protease and phosphatase inhibitors. Fractionation and mitochondrial isolation details are found in Supplementary Data S1. After 30 minutes of incubation on ice, lysates were centrifuged (13,000 × *g*, 20 minutes, 4°C), and protein concentration in the supernatant was determined using the DC Protein Assay Kit (Biorad) on a Synergy HT Plate Reader (BioTek). Then, 10 μg protein per sample was loaded and separated on NuPAGE 4 to 12% Bis‐Tris gels (Invitrogen). Proteins were transferred to a nitrocellulose membrane (Protran) and detected with various antibodies, as listed in Supplementary Data S1.

### mt‐mKeima Assay and Confocal Imaging

The mt‐mKeima assay and confocal microscopy was performed as previously described.^30^ Further details are described in Supplementary Data S1.

### Statistical Analysis

Statistical analyses were performed in R software (RStudio). Detailed methods are described in Supplementary Data S1.

### Study Approval

The study was approved by the ethics committee of the University of Lübeck (04–155 and 05–030), and all participants signed written informed consent before they participated in the study.

## Results

### Biallelic *PRKN* ^delEx2^ Variant Carriers Develop Parkinson's Disease at a Later Age Compared to Patients With PARK‐ *PRKN* Carrying Other Variants

**FIGURE 2:**
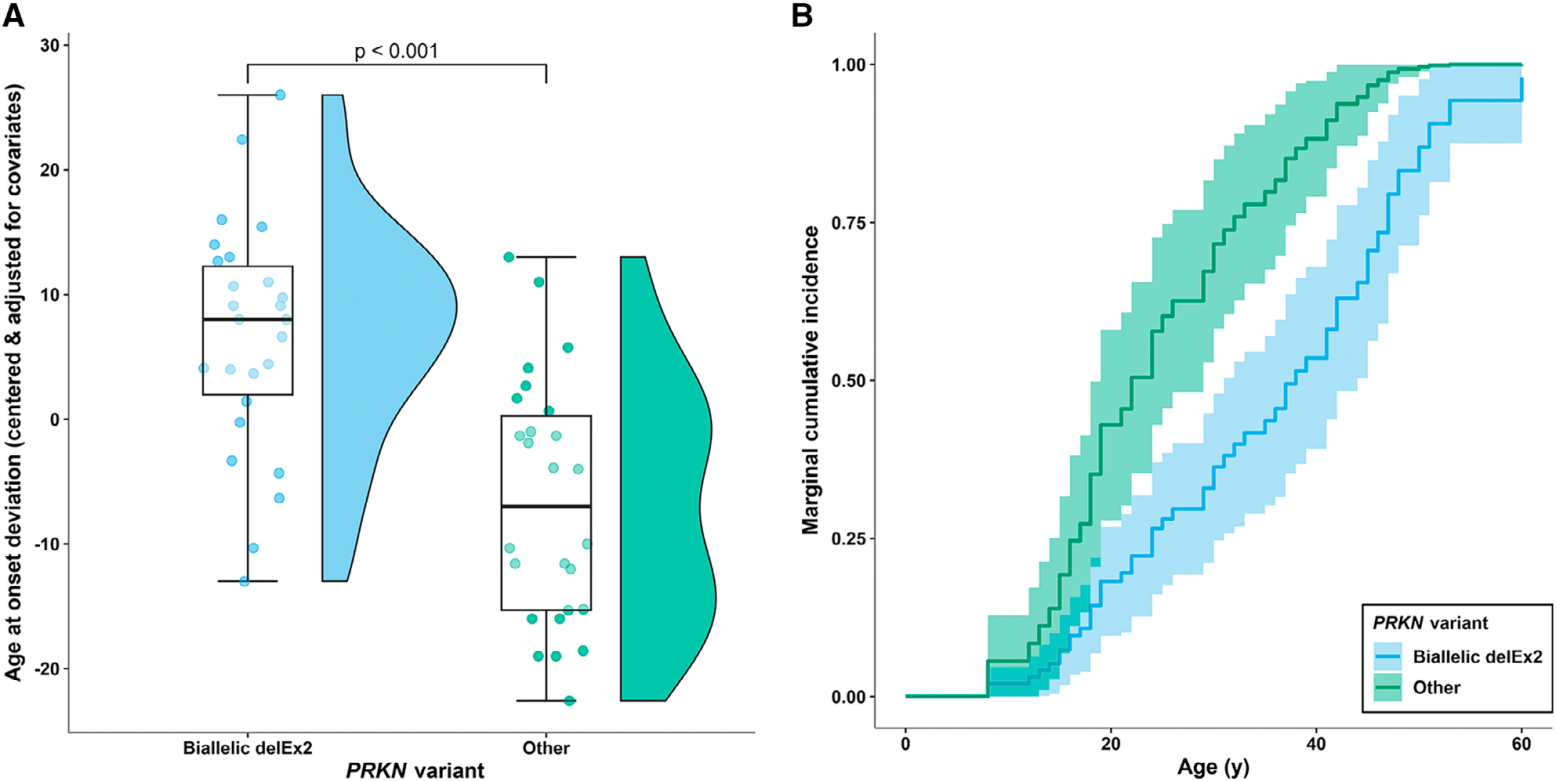
Later age at onset in biallelic *PRKN* ^delEx2^ carriers. (A) Partial residual plot highlighting the impact of a biallelic *PRKN* ^delEx2^ variant on AAO compared with matched carriers of other pathogenic *PRKN* variants with an estimated average marginal effect of 13.22 years (*t*(45) = 4.88, *p* < 0.001). Covariates corrected for in the underlying linear regression model were sex and country of origin. (B) A Cox proportional hazards model to estimate the marginal cumulative incidence and HR for biallelic *PRKN* ^delEx2^ variant carriers and matched variant carriers (HR = 0.36, SE = 0.26, *z* = −3.79, *p* < 0.001). Sample size = 26 per group. The significance threshold was set to *p* = 0.05. Whiskers extend to the largest and smallest values no further than 1.5 * IQR from the hinge. Reported standard errors are cluster robust. AAO = age at onset; HR = hazard ratio; IQR = interquartile range. [Color figure can be viewed at www.annalsofneurology.org]

A review of the MDSGene database suggested a homozygous *PRKN* ^delEx2^ variant causes PD with a later AAO compared with carriers of other *PRKN* variants, as indicated by a higher median AAO (39.5 vs 31 years) and the identification of an unaffected 86‐year‐old individual.^21^ To explore this further, we contacted the principal investigators of several larger patient cohorts. We analyzed 26 *PRKN* ^delEx2^ carriers and 26 carriers of other *PRKN* variants with detailed demographic and clinical data, who were matched on the propensity score, including the covariates sex and country of origin. We fitted a linear regression model to predict AAO, using a binary indicator variable for homozygous *PRKN* ^delEx2^ variant status with sex and country of sample origin as covariates. Our model indicates an association between homozygous variant status and AAO predicting an AAO difference of 13.22 years compared with paired carriers of other *PRKN* variants (*p* < 0.001; Fig 2A). A Cox proportional hazards model estimated a 64% reduction in hazard ratio for these carriers (*p* < 0.001; Fig 2B).

Additionally, we classified all patients more generally by variant, located either upstream or downstream of the internal TIS, starting at position c.238, and thus for its potential to translate the Parkin^Δ1–79^ protein (see below). *PRKN* ^delEx1^ variants were not classified as upstream because they affected the promoter and resulted in the loss of the canonical translation start site. Associations with AAO for upstream variants were determined by extending the *PRKN* ^delEx2^ linear model, and without prior propensity score matching, as the matched data for these additional binary indicators did not pass model assumptions. Unlike biallelic *PRKN* ^delEx2^ variants, variants upstream of the internal TIS were not associated with AAO, whether present on one (*p* = 0.216) or both alleles (*p* = 0.654), compared with downstream variants. Similarly, single *PRKN* ^delEx2^ alleles in a total of 20 compound heterozygous patients (Supplementary Table S4) showed no association with AAO (*p* = 0.515). Variant impact stratification (missense as “moderate” and others as “high”) revealed no additional associations with AAO.

### In Silico Translation Initiation Prediction for Parkin^Δ1^ ^−79^

We predicted Parkin^Δ1–79^ translation potential by scoring TIS in various *PRKN* transcripts using TIS Transformer (Table 2).^31^ Wild‐type *PRKN* showed the highest probability for the canonical TIS (0.94) versus the internal TIS at c.238 (<0.01). Conversely, the internal TIS in the *PRKN* ^delEx2^ transcript scored the highest (0.96), supporting preferential translation of Parkin^Δ1–79^. In the *PRKN* ^c.100_101insC^ line, the canonical TIS probability was 0.11, and an out‐of‐frame TIS at position c.32 was 0.57. The internal TIS in *PRKN* ^c.100_101insC^ was scored low as in wild‐type *PRKN* (<0.1). Notably, the reading frame restores at c.100 for the out‐of‐frame TIS at position c.32 due to the frameshift. This should result in the Parkin isoform that is 2.5 kDa smaller than the full‐length Parkin and differs from the wild‐type Parkin in 25 amino acids at the N‐terminus. This most likely leads to protein instability, as a faint band of the appropriate size observed in some Western blots of *PRKN* ^c.100_101insC^ cells may match this prediction (Fig 4A). Notably, a hypothetical variant disrupting the canonical TIS (c.2T>C) increased internal TIS probability to 0.89.

**Table 2:** In Silico TIS Prediction by TIS Transformer

Transcript ID	Transcript Length	Abs. TIS Position	c. Position	Probability Score	Start Codon	Stop Position	Stop Codon	Protein Length
*PRKN* wildtype	4,178	12	−87	0.0018	CAG	1,493	TAG	494
*PRKN* wildtype	4,178	99	1	**0.9391**	ATG	1,493	TAG	465
*PRKN* wildtype	4,178	336	238	*0.0080*	ATG	1,493	TAG	386
*PRKN* delEx2	4,014	56	−43	0.0132	ATG	172	TGA	39
*PRKN* delEx2	4,014	172	238	***0.9637***	ATG	1,329	TAG	386
*PRKN* delEx2	4,014	508	574	0.0016	ATG	1,329	TAG	274
*PRKN* c.100dupC	4,179	12	−87	0.0041	CAG	212	TGA	67
*PRKN* c.100dupC	4,179	99	1	0.1157	ATG	212	TGA	38
*PRKN* c.100dupC	4,179	130	32	**0.5677**	ATG	1,494	TAG	455
*PRKN* c.100dupC	4,179	337	238	*0.0817*	ATG	1,494	TAG	386
*PRKN* c.100dupC + c.33T>A	4,179	12	−87	0.0046	CAG	212	TGA	67
*PRKN* c.100dupC + c.33 T>A	4,179	56	−43	0.0015	ATG	154	TGA	33
*PRKN* c.100dupC + c.33T>A	4,179	99	1	0.2303	ATG	212	TGA	38
*PRKN* c.100dupC + c.33T>A	4,179	337	238	***0.8589***	ATG	1,494	TAG	386
*PRKN* c.2T>C	4,178	12	−87	0.1230	CAG	1,493	TAG	494
*PRKN* c.2T>C	4,178	56	−43	0.0012	ATG	154	TGA	33
*PRKN* c.2T>C	4,178	130	32	0.0015	ATG	225	TGA	32
*PRKN* c.2T>C	4,178	219	121	0.0024	TTG	1,493	TAG	425
*PRKN* c.2T>C	4,178	336	238	***0.8935***	ATG	1,493	TAG	386

*Note*: The highest probability score per transcript is presented in bold. Probability scores for the internal TIS are presented in italics.

TIS = translation initiation site.

### Overexpressed Parkin^Δ1^ ^−79^ Variant Shows Residual Activity

Previous studies demonstrated that Parkin^Δ1–79^ expressed from an internal TIS at position c.238 retains partial function.^16^, ^17^, ^19^, ^20^, ^32^ We hypothesized that later AAO in *PRKN* ^delEx2^ carriers results from residual Parkin^Δ1–79^ function, as the Exon 2 deletion shifts the internal TIS in Exon 3 closer to the 5′ regulatory region (see Fig 1B), likely promoting enhanced translation efficiency as predicted in silico.

**FIGURE 3:**
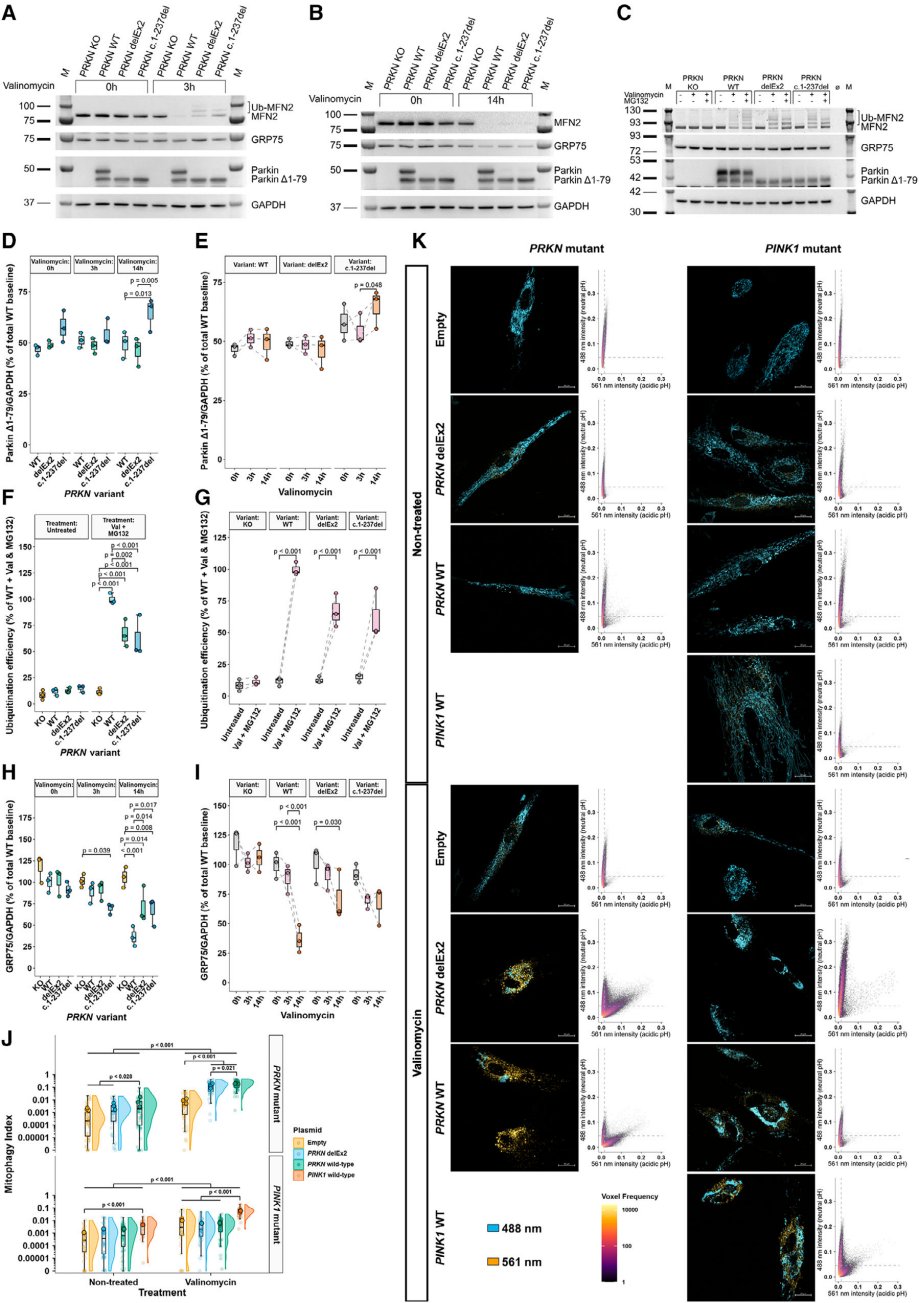
Overexpression of 3 *PRKN* constructs in a *PRKN* knockout neuroblastoma cell model and PARK‐*PRKN* patient fibroblasts highlights Parkin^Δ1–79^ activity. (A, B) Western blot analysis of MFN2, GRP75, and Parkin in *PRKN*‐KO SH‐SY5Y neuroblastoma cells overexpressing *PRKN* ^wt^, *PRKN* ^delEx2^, or *PRKN* ^c.1–237del^, a shortened construct, starting directly at the internal translation initiation site. Mitochondrial depolarization was induced by 1 μM valinomycin for 3 hours (A) and 14 hours (B), respectively. (C) Reproduction of (A), with inclusion of MG132 proteasome inhibitor treatment to allow accurate calculation of ubiquitination efficiencies given the rapid MFN2 turnover. Signals were normalized against GAPDH. (D, E) Parkin^Δ1–79^ expression was comparable between all overexpression models (D) and remained stable after prolonged valinomycin treatment (E). (F, G) Between (F) and within‐group (G) differences in Parkin ubiquitination efficiencies of MFN2. (H, I) Differences in GRP75 levels between groups (H) and over time (I). Sample size = 3 from independently repeated experiments across three cell passages. (J) Super plot showing mitophagy differences in PARK‐*PRKN* and PARK‐*PINK1* patient fibroblasts transfected to express *PRKN* ^wt^, *PRKN* ^delEx2^, or *PINK1* ^wt^, or with an empty vector, following mitochondrial depolarization. A total of 676 cells (faint data points) were quantified across 3 different PARK‐*PRKN* patient fibroblast lines and one PARK‐*PINK1* cell line (opaque data points, arithmetic mean). (K) Maximum projections of mt‐mKeima confocal images of non‐treated and 12 hours of depolarized fibroblasts and corresponding voxel frequencies. Total voxels were down sampled to 500.000 for visualization. Background voxels were filtered out using the same thresholds applied for background signal subtraction. Dashed lines mark the crosshair coordinates chosen for mitophagy index calculation. The significance threshold was set to *p* = 0.05. Whiskers extend to the largest and smallest values no further than 1.5* IQR from the hinge. Pairwise comparisons of linear mixed effects model (D–I) or generalized mixed effects model (J)‐derived estimated marginal means were Holm‐adjusted. IQR = interquartile range. [Color figure can be viewed at www.annalsofneurology.org]

We investigated Parkin levels and ligase activity in *PRKN*‐KO (genome‐edited *PRKN* ^delEx3^) neuroblastoma cells overexpressing *PRKN* ^delEx2^, *PRKN* ^c.1_237del^ (a direct internal TIS construct), or wild‐type *PRKN* following valinomycin treatment (Fig 3). We analyzed Parkin^Δ1–79^ levels in these lines (see Fig 3A, B, D, E). Under basal conditions, levels of Parkin^Δ1–79^ were comparable among all cell lines but showed an increase in *PRKN* ^c.1–237del^ cells after prolonged depolarization (*p* < 0.005; see Fig 3D, E). Total Parkin expression (full‐length Parkin + Parkin^Δ1–79^) was 41 to 62% lower in cells expressing truncated constructs versus wild‐type (Supplementary Fig S3A).

Parkin ligase activity was assessed via ubiquitinated‐to‐non‐ubiquitinated MFN2 ratio. Cells were treated with valinomycin for mitochondrial depolarization and the proteasome inhibitor MG132 for 3 hours to protect Ub‐MFN2 from proteasomal degradation (see Fig 3C, F, G). As expected, ubiquitination efficiency was highest in cells overexpressing wild‐type Parkin (*p* < 0.002; see Fig 3F). Both *PRKN* ^delEx2^ and *PRKN* ^c.1–237del^ cells exhibited a 33% and 38% lower ubiquitination efficiency compared with *PRKN* ^wt^ cells, respectively, while maintaining 5.9‐ and 5.5‐fold higher efficiencies than *PRKN* ^KO^ cells (*p* < 0.001). Ubiquitination efficiency was increased in all overexpression models after mitochondrial depolarization (*p* < 0.001), but not in the non‐transduced cells (*p* = 0.705; see Fig 3G).

To further assess the removal of depolarized mitochondria, protein levels of the mitochondrial matrix protein GRP75 were measured (see Fig 3H, I). Compared with the non‐transduced cells, GRP75 levels were significantly reduced in *PRKN* ^c.1–237del^ cells upon 3 hours of valinomycin treatment (*p* = 0.039) and reduced in all Parkin overexpression models following 14 hours of mitochondrial depolarization (*p* < 0.014; see Fig 3H). Upon prolonged valinomycin treatment, GRP75 levels decreased in all cells (*PRKN* ^wt^ [*p* < 0.001], *PRKN* ^delEx2^ [*p* = 0.030], and *PRKN* ^c.1–237del^, [borderline significant, *p* = 0.061]; see Fig 3I).

For a more direct endpoint measurement of *PINK1*/Parkin‐dependent mitophagy, fibroblasts from PARK‐*PRKN* and PARK‐*PINK1* patients expressing mt‐mKeima were transfected either with empty vector (EV) or with vectors expressing either *PRKN* ^wt^ or *PRKN* ^delEx2^. *PINK1*‐PD fibroblasts were additionally transfected with *PINK1* ^wt^ (see Fig 3J, K) to rescue the phenotype. In PARK‐*PRKN* fibroblasts, expression of *PRKN* ^delEx2^ showed increased mitophagy compared with both PARK‐*PRKN* EV controls (*p* < 0.001) and *PINK1*‐deficient cells overexpressing *PRKN* ^delEx2^ or *PINK1* ^wt^ (*p* < 0.001). Mitophagy was lower in fibroblasts expressing *PRKN* ^delEx2^ compared to *PRKN* ^wt^ (*p* = 0.021). In *PINK1*‐PD fibroblasts, as expected, valinomycin‐induced mitophagy was increased only in cells overexpressing *PINK1* ^wt^ (*p* < 0.001), but not *PRKN* ^wt^ nor *PRKN* ^delEx2^, because mitochondrial translocation of Parkin is dependent on *PINK1*. Notably, Valinomycin treatment led to an increase in mitophagy in all lines compared to non‐treated cells, indicating *PINK1*/Parkin‐independent mitophagy.

### Parkin^Δ1^ ^−79^ is Expressed in SH‐SY5Y Cells Carrying a Frameshift Variant Upstream of the Internal TIS and Displays Residual Mitochondrial Translocation

**FIGURE 4:**
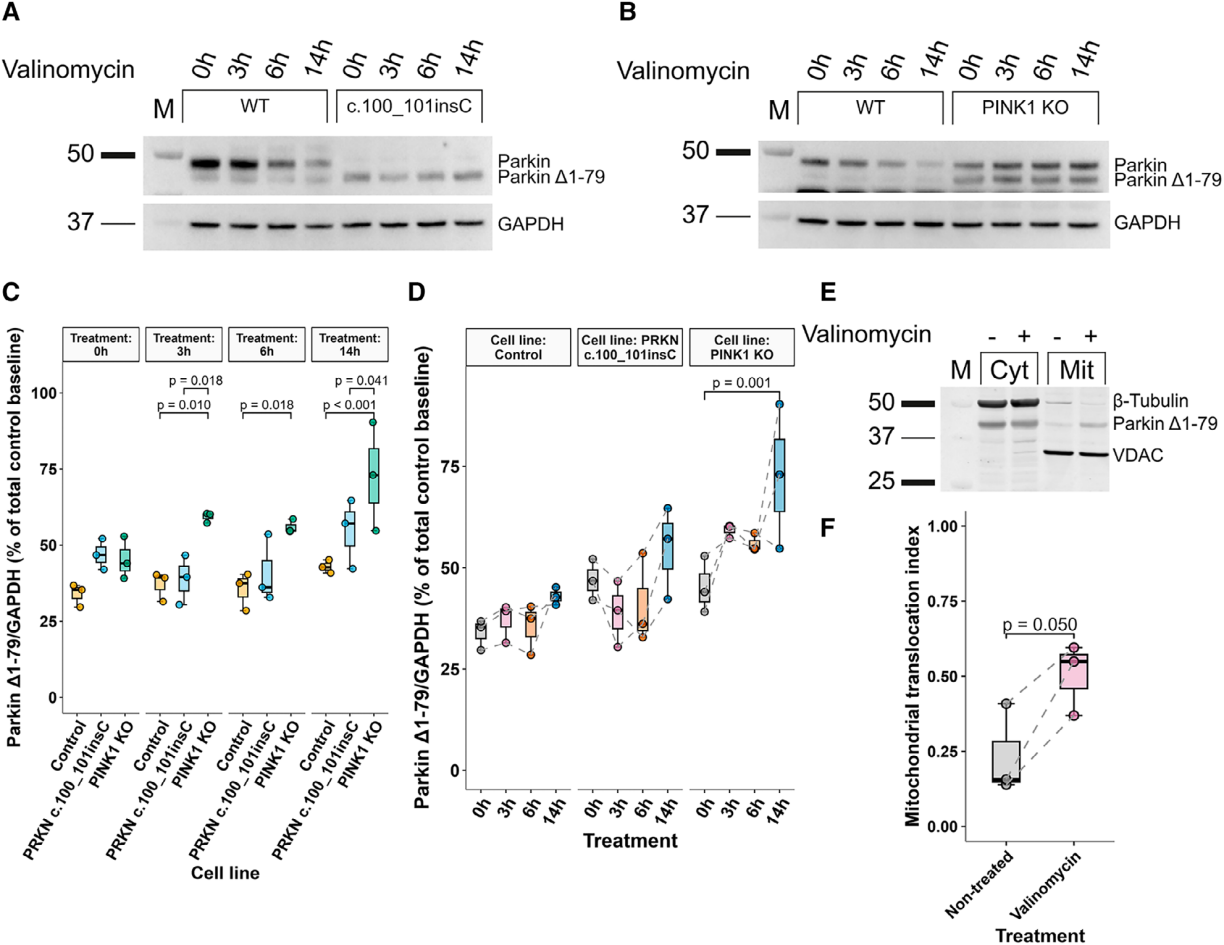
Endogenous Parkin expression in wild‐type, *PRKN* ^c.100_101insC^, and *PINK1* knockout neuroblastoma cells and subcellular localization in *PRKN* ^delEx2^ smNPCs. (A, B) Western blot analysis of endogenous Parkin in wild‐type, *PRKN* ^c.100_101insC^ (A), and *PINK1*‐KO (B) SH‐SY5Y neuroblastoma cells following mitochondrial depolarization with 1 μM valinomycin across multiple time points. The c.100_101insC *PRKN* variant introduces a frameshift in Exon 2 of *PRKN*. Expression of Parkin^Δ1–79^, initiated upstream of the frameshift variant, remains intact. Signals were normalized against GAPDH. (C, D) Parkin^Δ1–79^ protein levels between groups (C) and over time (D). (E) Assessment of Parkin translocation after 1 μM valinomycin‐induced mitochondrial depolarization by fractionation and subsequent western blot analysis. Cytosolic and mitochondrial fractions were isolated from proband‐derived biallelic *PRKN* ^delEx2^ carrier smNPCs. (F) Translocation index of Parkin^Δ1–79^ in non‐treated smNPCs compared with valinomycin‐treated cells. Sample size = 3 from independently repeated experiments across three cell passages. The significance threshold was set to *p* = 0.05. Whiskers extend to the largest and smallest values no further than 1.5*IQR from the hinge. Pairwise comparisons of linear mixed effects model derived estimated marginal means (C, D) were Holm‐adjusted. smNPC = small molecule neuronal precursor cell; IQR = interquartile range. [Color figure can be viewed at www.annalsofneurology.org]

We hypothesized that *PRKN* variants upstream of the internal TIS, absent cis‐regulatory disruption, would preserve or even increase Parkin^Δ1–79^ levels due to shifted TIS favorability. To test this, we analyzed endogenous Parkin levels in wild‐type, *PRKN* ^c.100_101insC^, and *PINK1* ^−KO SH‐SY5Y^ cells under basal conditions and following valinomycin treatment (Fig 4A–D).

As expected, no full‐length Parkin was detected in *PRKN* ^c.100_101insC^ (see Fig 4A, B), whereas Parkin^Δ1–79^ remained expressed. Interestingly, levels of Parkin^Δ1–79^ were higher in *PRKN* ^c.100_101insC^ cells when compared with wild‐type cells (see Fig 4D), suggesting that frameshift variants upstream of internal TIS enhance translation from the internal TIS.

Upon valinomycin treatment, levels of full‐length Parkin gradually decreased with prolonged depolarization only in wild‐type cells (*p* = 0.043), indicating continuous autoubiquitination of full‐length Parkin (Supplementary Fig S4D). In contrast, Parkin^Δ1–79^ levels remained comparatively stable in both wild‐type and *PRKN* ^c.100_101insC^ cells (see Fig 4C). In PINK1‐KO cells, levels of both Parkin forms combined and Parkin^Δ1–79^ alone instead increased up to 80% (*p* < 0.001) and 62% (*p* = 0.002), respectively, with prolonged depolarization (see Fig 4D and Supplementary Fig S4B) due to impaired *PINK1*‐dependent mitochondrial translocation of Parkin and its autoubiquitination in *PINK1*‐deficient cells.

To assess mitochondrial translocation of the Parkin^Δ1–79^ proteoform, we quantified endogenous Parkin in cytosolic and mitochondrial fractions in hiPSC‐derived smNPCs derived from the unaffected 86‐year‐old homozygous *PRKN* Exon 2 deletion carrier (Fig 4E, F). Following 6 hours of valinomycin treatment, Parkin^Δ1–79^ partially translocated to the mitochondrial fraction as shown by an increased translocation index compared to non‐treated smNPCs (*p* = 0.0497; see Fig 4F). We demonstrated mitochondrial translocation of Parkin^Δ1–79^ in SH‐SY5Y cells (see Supplementary Fig S4E, S4H). The wild‐type cells displayed initial mitochondrial Parkin recruitment, followed by a decrease during prolonged depolarization due to Parkin's autoubiquitination and degradation (see Supplementary Fig S4G–I). In *PINK1*‐KO cells, we observed no Parkin translocation (Supplementary Fig S4F).

### Residual Parkin Function in *PRKN* ^delEx2^ Patient‐Derived Midbrain Dopaminergic Neurons

To validate findings in more relevant, patient‐derived neuronal cell models, we analyzed 3 independent differentiations of iPSC‐derived dopaminergic neurons (iDNs) from 2 patients with PARK*‐PRKN* carrying a homozygous deletion in either Exon 2 or Exon 7, two isogenic pairs carrying a biallelic Exon 2 deletion, an isogenic pair carrying a biallelic Exon 3 deletion, and 2 healthy controls.

**FIGURE 5:**
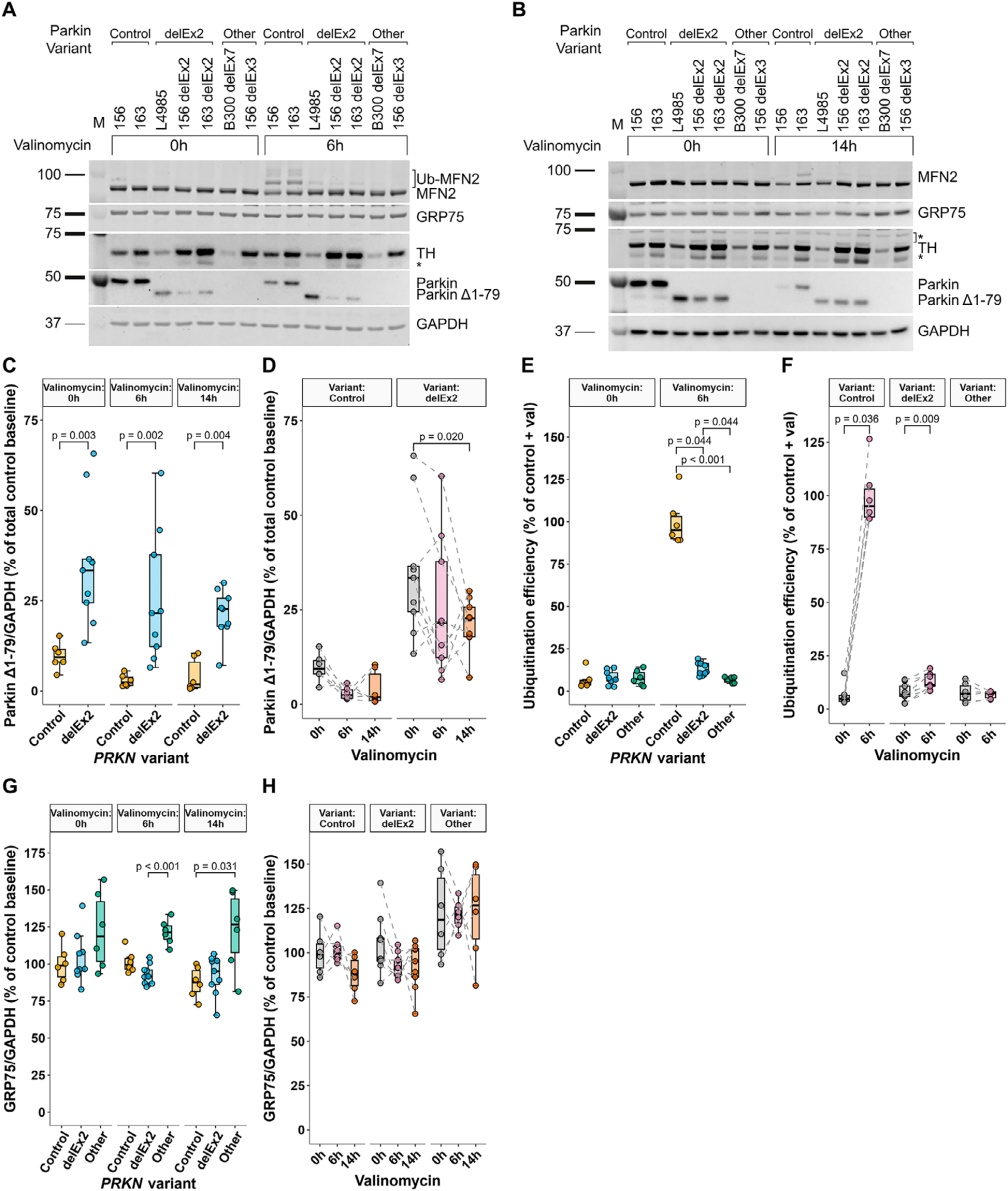
Residual endogenous Parkin function and increased Parkin^Δ1–79^ levels in biallelic *PRKN* ^delEx2^ patient‐derived dopaminergic midbrain neurons. (A, B) Western blot analysis of MFN2, GRP75, TH, and Parkin in hiPSC‐derived midbrain dopaminergic neurons of healthy controls (n = 6), iso and heterogenic biallelic *PRKN* ^delEx2^ carriers (n = 9), as well as carriers of other biallelic *PRKN* exon deletions (n = 6) following 1 μM valinomycin treatment for 6 hours (A) and 14 hours (B). Signals were normalized against GAPDH. (C, D) Parkin^Δ1–79^ expression differences between groups (C) and expression changes with increased mitochondrial depolarization periods (D). (E, F) Differences in MFN2 ubiquitination between *PRKN* variant carriers (E) and MFN2 ubiquitination change over time (F). (G, H) Comparison of GRP75 expression between *PRKN* variant carriers (G) and over prolonged valinomycin treatment (H). Sample sizes represent a combination of individual patient‐derived neurons and independently repeated experiments across 3 differentiations. The significance threshold was set to *p* = 0.05. Whiskers extend to the largest and smallest values no further than 1.5*IQR from the hinge. Post hoc multiple comparisons via Dunn tests (E, G) and Durbin‐Conover tests (D) were Holm‐adjusted. hiPSC = human‐induced pluripotent stem cell; IQR = interquartile range. [Color figure can be viewed at www.annalsofneurology.org]

The neurons were analyzed under basal conditions and upon valinomycin‐induced mitochondrial depolarization. *PRKN* ^delEx2^ carriers showed higher Parkin^Δ1–79^ levels compared with controls (*p* < 0.004; Fig 5A–C). Parkin^Δ1–79^ levels decreased with prolonged mitochondrial depolarization in carriers of biallelic *PRKN* ^delEx2^ (*p* = 0.032) with a similar, albeit nonsignificant trend in controls (*p* = 0.135; Fig 5D). Whereas total Parkin was higher under basal conditions in healthy controls (*p* = 0.011), Parkin^Δ1–79^ was more stable in *PRKN* ^delEx2^ carriers after 14 hours of mitochondrial depolarization (*p* = 0.029; Supplementary Fig S5A, S5B). Other *PRKN* deletion carriers (Exon 3 or 7) showed neither full‐length Parkin nor Parkin^Δ1–79^. Importantly, *PRKN* mRNA levels in patients with *PRKN* ^delEx2^ were comparable with controls, whereas other deletion carriers showed 40 to 50% reductions (Supplementary Fig S5C), most likely due to the nonsense‐mediated decay.

Endogenous Parkin's ubiquitin ligase activity in *PRKN* ^delEx2^ carriers was assessed by measuring levels of non‐modified and ubiquitinated MFN2 in iDNs (see Fig 5A–D) and human dermal fibroblasts (Supplementary Fig S6A–D) upon valinomycin treatment for 6 hours. As expected, iDNs derived from healthy controls exhibited significantly higher Parkin ubiquitination efficiency compared to both *PRKN* ^delEx2^ neurons and neurons carrying other exon deletions (*p* < 0.044). Crucially, iDNs from biallelic *PRKN* ^delEx2^ carriers had higher ubiquitination efficiency than those from carriers of other *PRKN* variants (*p* = 0.044) and showed increased activity upon depolarization (*p* = 0.009), confirming residual function (Fig 5E, F). Analogous experiments in human dermal fibroblast cultures replicated these findings (see Supplementary Fig S6A–D).

GRP75 quantification indicated a reduction in *PRKN* ^delEx2^ carriers after 6 hours (*p* < 0.001) and healthy controls after 14 hours (*p* = 0.031) of valinomycin treatment when compared with carriers of other *PRKN* variants (Fig. 5G, H).

### Molecular Glue BIO‐2007817 Increases Endogenous Parkin ^Δ1–79^ Activity

Based on reports that Tetrahydropyrazolo‐pyrazine (THPP) compounds can activate Ubl‐deficient Parkin variants, we hypothesized the molecular glue BIO‐2007817 would boost endogenous Parkin^Δ1–79^ activity in *PRKN* ^delEx2^ carriers.^22^, ^23^

**FIGURE 6:**
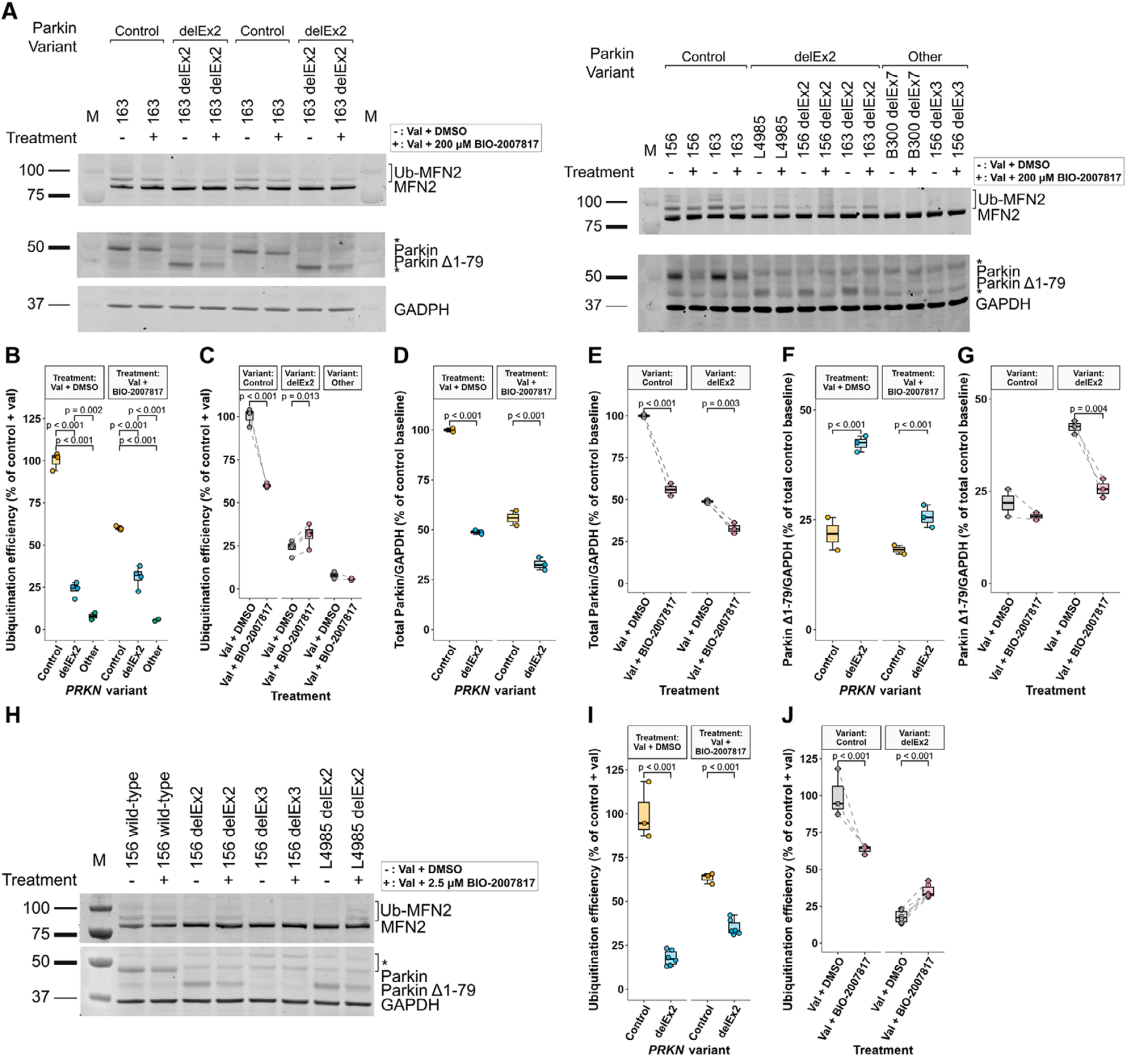
BIO‐2007817 partially rescues endogenous Parkin function in biallelic *PRKN* ^delEx2^ patient‐derived dopaminergic midbrain neurons. (A) Western blot analysis of MFN2 and Parkin in hiPSC‐derived dopaminergic midbrain neurons of healthy controls (n = 3), carriers of biallelic *PRKN* ^delEx2^ (n = 4), or other biallelic exon deletion variants (n = 2) following treatment with an allosteric Parkin activator. All neurons were preincubated for 1 hour with either DMSO or 200 μM BIO‐2007817, followed by adding 1 μM valinomycin for a further 6 hours. Signals were normalized against GAPDH. (B) Between‐group differences of MFN2 ubiquitination efficiency with and without BIO‐2007817 treatment. (C) Effect of BIO‐2007817 on MFN2 ubiquitination efficiency per *PRKN* variant group. (D, E) Total Parkin (full‐length Parkin + Parkin^Δ1–79^) protein level differences between groups (D) and its BIO‐2007817 induced change (E). (F, G) Variation of Parkin^Δ1–79^ expression between (F) and within groups (G). As cross‐blot normalization was not feasible for the analysis of Parkin levels here, only the right blot was included in the analysis. Sample sizes represent a combination of individual patient‐derived neurons and independently repeated experiments across two differentiations. (H–J) Validation of BIO‐2007817 boosted Parkin^Δ1–79^ activity in smNPCs using a more pharmacologically relevant concentration of 2.5 μM. The significance threshold was set to *p* = 0.05. Whiskers extend to the largest and smallest values no further than 1.5*IQR from the hinge. Pairwise comparisons of linear mixed effects model derived estimated marginal means (C–F) were Holm‐adjusted. hiPSC = human‐induced pluripotent stem cell; IQR = interquartile range; smNPC = small molecule neuronal precursor cell [Color figure can be viewed at www.annalsofneurology.org]

To test this, we determined effects of BIO‐2007817 pretreatment on Parkin levels and Parkin's ubiquitination efficiency by measuring ubiquitination of MFN2 after 6 hours of mitochondrial depolarization (Fig 6A). As established previously, ubiquitination efficiency was the highest in controls, intermediate in *PRKN* ^delEx2^ carriers, and lowest in other *PRKN* deletion carriers (*p* < 0.002; Fig 6B). Crucially, 200 μM BIO‐2007817 increased ubiquitination efficiency by 30% in *PRKN* ^delEx2^ carriers (*p* = 0.014), restoring it to 31% of control levels (Fig 6C). Conversely, THPP treatment induced reduction in ubiquitination efficiency in healthy controls (*p* < 0.001).

Levels of total Parkin decreased with THPP treatment in both groups (*p* < 0.003; Fig 6D, E). In DMSO‐treated cells, levels of Parkin^Δ1–79^ were overall higher in *PRKN* ^delEx2^ carriers compared with healthy controls (*p* < 0.029; Fig 6F), consistent with our previous findings (see Fig 5E). With THPP treatment, Parkin^Δ1–79^ significantly decreased in *PRKN* ^delEx2^ (*p* = 0.004), but not in healthy control neurons (*p* = 0.260; Fig 6G), suggesting enhanced turnover of Parkin^Δ1–79^ concurrent with increased Parkin's activity.

To rule out concentration‐dependent off‐target effects potentially caused by the high concentration used for maximum target engagement (200 μM), we repeated this experiment using a more pharmacologically relevant concentration of 2.5 μM in smNPCs derived from a healthy control, an isogenic biallelic *PRKN* ^delEx2^ line, and from the *PRKN* ^delEx2^ biallelic carrier (Fig 6H). Consistent with our findings in fully differentiated iDNs, smNPCs expressing Parkin^Δ1–79^ showed a significant increase in ubiquitination efficiency upon BIO‐2007817 pretreatment and subsequent mitochondrial depolarization (*p* = 0.004; Fig 6I, J). Here too, ubiquitination efficiency in healthy controls treated with BIO‐2007817 decreased to 63% of smNPCs treated with valinomycin only (*p* < 0.001), indicating an inhibitory effect on endogenous full‐length Parkin even at a lower dose optimized in previous cell models.^22^

## Discussion

With this translational study, we link the later AAO of patients with PD carrying a biallelic Ex2 deletion to elevated levels of partially active truncated Parkin^Δ1–79^ variant. Notably, we provide the first evidence for allosteric modulation of endogenous Parkin^Δ1–79^ in a patient‐derived in vitro PD model by a THPP compound, which partially restores ubiquitination activity. These findings imply: (i) a *PRKN* ^delEx2^‐specific later PARK*‐PRKN* disease onset compared with carriers of other variants, (ii) opportunities for personalized counseling and treatment strategies in patients with residual Parkin^Δ1–79^ expression, and (iii) important considerations for *PRKN*‐variant research and gene knockout strategies that account for residual functionality of truncated Parkin^Δ1–79^.

Using aggregated patient data,^6^, ^24^, ^25^ we investigated whether elevated Parkin^Δ1–79^ expression in vitro, particularly in biallelic carriers of *PRKN* ^delEx2^ variants, would translate to a specific clinical manifestation of PD by affecting the median AAO. Our linear model on propensity score‐matched patients with PARK*‐PRKN* estimated a later AAO in biallelic *PRKN* ^delEx2^ variant carriers compared with carriers of other variants by 13.22 ± 2.71 years on average, likely linked to increased Parkin^Δ1–79^ levels and partial function. Of note, biallelic *PRKN* ^delEx2^ carriers also displayed a wide AAO range (13–60 years), warranting further research into whether this variation reflects differences in Parkin^Δ1–79^ levels. Potential modifiers include cis‐ and trans‐regulatory elements, ribosomal efficiency at the internal TIS, additional rare variants, polygenic risk, and environmental or comorbid factors.^33^ Notably, previous analysis of the asymptomatic *PRKN* ^delEx2^ carrier functionally characterized here revealed elevated NIX expression, which mediates alternative Parkin‐independent mitophagy.^34^ Dual compensatory mechanisms, elevated NIX and residual Parkin^Δ1–79^ function, may synergistically preserve mitochondrial homeostasis and contribute to disease protection in this remarkable case.

Interestingly, a single *PRKN* ^delEx2^ variant and other pre‐iTIS variants were not associated with a later AAO. In compound heterozygous *PRKN* ^delEx2^ carriers, we initially expected an intermediate effect, but the smaller sample size and potential confounders may have limited the detection of such a shift. Our findings instead suggest that the gene dosage of Parkin^Δ1–79^‐competent alleles, such as *PRKN* ^delEx2^, is crucial in affecting AAO. Uniquely, the *PRKN* ^delEx2^ variant decreases the distance between the regulatory 5′‐untranslated region and the internal TIS, likely facilitating increased Parkin^Δ1–79^ levels. For other pre‐iTIS variants, translation initiation may primarily occur at the canonical start codon, yielding misfolded and unstable protein.^35^ Consequently, lower levels of internal ATG initiation likely result in reduced Parkin^Δ1–79^ quantity compared with *PRKN* ^delEx2^ carriers. Thus, our finding complements previous research on genotype–phenotype correlations.^7^

Whereas Parkin^Δ1–79^ presence and partial function of similar ΔUbl variants using overexpression models have been observed previously,^16^, ^17^, ^18^, ^19^, ^20^, ^22^, ^32^, ^36^ its endogenous activity, stability, and regulation in PD patient‐derived material remained unclear. Western blot quantification revealed increased truncated Parkin^Δ1–79^ levels in cells carrying an endogenous biallelic *PRKN* ^delEx2^ variant but not in a homozygous *PRKN* ^c.100_101insC^ cell model. These patterns, together with high internal TIS score prediction in silico, indicate that closer iTIS proximity to the transcripts’ 5′ end in *PRKN* ^delEx2^ carriers may enhance Parkin^Δ1–79^ translation.

Unlike full‐length Parkin, Parkin^Δ1–79^ quantities remained relatively stable after prolonged mitochondrial depolarization. Full‐length Parkin self‐regulates by autoubiquitination and subsequent proteasomal degradation.^37^ Reduced ubiquitin ligase activity and mitochondrial recruitment of Parkin^Δ1–79^ may impair autoubiquitination. Furthermore, previous affinity purification‐mass spectrometry experiments suggested association of a ΔUbl‐Parkin fragment, similar to Parkin^Δ1–79^, with the outer mitochondrial membrane, but not with the proteasome.^38^ Thus, lack of the Ubl‐domain may impair proteasomal association and degradation of Parkin^Δ1–79^, stabilizing it.

Previous functional assessments of Parkin^Δ1–79^ and closely related ΔUbl‐Parkin fragments focused on assays relying on overexpression experiments.^16^, ^17^ Complementing these findings, we provide multi‐level evidence for endogenous Parkin^Δ1–79^ function in patient‐derived neuronal models of an asymptomatic biallelic *PRKN* ^delEx2^ carrier^21^ and CRISPR‐Cas9‐edited isogenic cell lines, as well as neuroblastoma cells harboring a pre‐internal TIS frameshift variant. Endogenous Parkin^Δ1–79^ is capable of substrate ubiquitination, *PINK1*‐dependent mitochondrial membrane translocation, and mitophagy initiation. This finding is in agreement with previous studies showing impaired but still detectable mitochondrial recruitment and activity of ΔUbl‐Parkin constructs.^10^, ^16^, ^39^ The residual activity occurs despite lacking the Ubl domain and, thus, its phosphorylation site, which facilitates efficient mitochondrial recruitment and Parkin activation via *PINK1* in full‐length Parkin.^40^ Previous studies demonstrated that phospho‐ubiquitin (pUb) alone is sufficient for the recruitment and activation of Parkin.^17^ Thus, mitochondrial pUb accumulation substitutes for the pUbl domain to displace RING2 and activate Parkin^Δ1–79^. Although experiments on purified proteins demonstrated that Ubl domain deletion can release Parkin from autoinhibition and enhance basal ubiquitination activity,^37^ we did not observe elevated basal levels of substrate ubiquitination or increased autoubiquitination of endogenous or exogenous Parkin^Δ1–79^. Although loss of the Ubl domain removes one critical autoinhibitory interaction, ΔUbl Parkin variants still require pUb binding to RING0 to drive the conformational change necessary for robust ubiquitin ligase activation.^17^ Without mitochondrial depolarization and *PINK1*‐mediated ubiquitin phosphorylation, endogenous Parkin^Δ1–79^ is likely to remain predominantly in a closed, inactive conformation, explaining the stability we observe. Of note, substrate ubiquitination by Parkin^Δ1–79^ was more comparable to full‐length Parkin in overexpression models than endogenous models, and the mt‐mKeima assay showed similar mitophagy levels for both variants when overexpressed. Here, high Parkin abundance likely compensates for reduced recruitment efficiency of Parkin^Δ1–79^. The presence and activity of Parkin^Δ1–79^ in patients with pre‐internal TIS *PRKN* variants adds complexity to variant interpretation and the generation of gene knockout strategies, as residual Parkin^Δ1–79^ can confound results and should be inactivated when complete loss‐of‐function is desired.

Recent progress in the development and characterization of THPP small molecule allosteric modulators of Parkin has demonstrated the capability of these compounds to selectively boost a Ubl‐independent Parkin activation mechanism.^22^, ^23^ We provide here the first evidence that BIO‐2007817 boosts activity and turnover of endogenous Parkin^Δ1–79^ in patient‐derived dopaminergic midbrain neurons. Notably, THPP treatment decreased ubiquitination activity in healthy controls at both saturating (200 μM) and pharmacologically relevant (2.5 μM) concentrations in cellular models,^22^ an opposing effect that has not been reported in exogenous expression models to date.^22^, ^23^ Given that THPP compounds occupy the same RING0 hydrophobic groove used by the ACT element in the fully active phosphorylated conformation, and that ACT and BIO‐2007817 compete for this site in vitro,^22^ it is conceivable that the compound interferes with optimal pUbl/ACT‐mediated activation of full‐length Parkin, thereby reducing its ubiquitination efficiency at endogenous expression levels. In contrast, Ubl‐deficient variants rely on pUb‐dependent activation at RING0 and lack a functional pUbl–ACT interface, so THPP binding in this groove predominantly acts as a molecular glue to enhance Rcat release and selectively boost their activity.^22^ This observation of variant‐selective modulation by BIO‐2007817 has critical implications for therapeutic development: whereas these compounds hold promise for patients with Ubl‐deficient *PRKN* variants, such as biallelic *PRKN* ^delEx2^ carriers, their use in individuals expressing Ubl‐competent Parkin would not only be ineffective, but potentially detrimental. Strict patient stratification based on *PRKN* genotype will be essential for clinical translation. Although our findings align with the established mechanism of BIO‐2007817 enhancing Parkin‐pUb interactions,^22^ alternative interpretations (for example, effects on deubiquitinases, substrate accessibility, or other E3 ligases) cannot be fully excluded. Dissecting the relative contributions of enhanced mitochondrial translocation versus increased ubiquitin ligase activity of Parkin^Δ1–79^ by THPP compounds remains an important objective for future studies. Crucially, our data support the potential of THPP compounds in future personalized treatment strategies for patients with residual Parkin^Δ1–79^ expression or defective Parkin Ubl domains. They may further benefit from deubiquitination inhibitors, such as MTX325, which, unlike THPPs, is already being examined in a phase 1 clinical trial.^41^

This study has some limitations. First, potentially confounding covariates included in propensity score matching and linear regression modeling for AAO were limited to sex and country of origin; broader availability of variables such as ancestry and comorbidities would likely improve the accuracy of outcome estimates. Second, a saturating concentration of BIO‐2007817 was initially tested, owing to limited iDN material. Although we validated key findings at a previously optimized concentration for cellular efficacy^22^ in a neuronal precursor model, the observed impairment of full‐length Parkin requires further mechanistic study to characterize potentially detrimental effects on Parkin variants with a functional Ubl‐domain.

In summary, our data provide translational evidence for residual Parkin function in a subset of patients with PARK*‐PRKN*. These findings offer additional functional insight into Parkin^Δ1–79^ and highlight a potential avenue for pharmacological intervention in patients with residual Parkin^Δ1–79^ expression. Further research should examine strategies to boost expression and responsiveness to THPP compounds as well as deubiquitination inhibitors in variant carriers beyond biallelic *PRKN* ^delEx2^. Moreover, how Parkin^Δ1–79^ level differences and their potential upstream causes affect the AAO range observed in biallelic *PRKN* ^delEx2^ carriers remain to be elucidated. Finally, besides *PRKN*, other disease‐linked genes may express truncated but partially functional proteins through internally initiated translation, affecting disease phenotypes.

## Author Contributions

C.K. and A.R. contributed to the conception and design of the study. A.H., K.L., M.F., P.J.M., E.J.V., T.K., H.Y., J.C.C., A.B., S.L., B.M., N.H., and C.M.S., contributed to the acquisition and analysis of data. A.H., K.L., C.K., and A.R. contributed to drafting the text or preparing the figures.

## Potential Conflicts of Interest

N.H. has received honoraria for lectures and speaker bureaus from Biogen, developer of BIO‐2007817 tested in this study. C.K. took part in a one‐time scientific meeting sponsored by Biogen (developer of BIO‐2007817 tested in this study), which focused on disease trajectories and biomarkers unrelated to this compound.

## Supporting information


**Supplementary Figure S1.** The hiPSC characterization. (C) Increased relative mRNA expression of the pluripotency markers GDF3. NANOG, OCT, and SOX2 in all newly generated and CRISPR‐Cas9 edited cell lines compared to Fibroblasts. (D) Increased relative mRNA expression of the ectodermal markers NCAM and PAX6, the mesodermal markers MSX1, MYH6, and RUNX1 as well as the endodermal markers GATA4 and SOX17 in embryoid bodies differentiated from all newly generated and CRISPR‐Cas9 edited cell lines compared to matched hiPSCs. (E) PCR amplification of *PRKN* Exon 1 to Exon 10 from iDN cDNA for all cell lines shown in main Figure 5. (F) Mycoplasma PCR test of all newly generated and CRISPR‐Cas9 edited cell lines. (G) PCR verifying the absence of Sendai virus reprogramming components for all newly generated and CRISPR‐Cas9 edited cell lines.
**Supplementary Figure S2.** Differentiation scheme to generate midbrain dopaminergic neurons from patient‐derived iPSCs. Neuronal precursor cells (NPCs) were plated on PDL/LA coated cell culture plates on day 20, as indicated in red. The iDNs were kept in culture until day 120. Created with BioRender.
**Supplementary Figure S3.** Total Parkin analysis between groups (A) and within groups (B) complementary to overexpression experiments of three *PRKN* constructs in a *PRKN* knockout neuroblastoma cell model in SH‐SY5Y cells shown in main Figure 3. Sample size: n = 3 from independently repeated experiments across three cell passages. The significance threshold was set to *p* = 0.05. Whiskers extend to the largest and smallest values no further than 1.5*IQR from the hinge. Pairwise comparisons of linear mixed effects model derived estimated marginal means were Holm‐adjusted.
**Supplementary Figure S4.** (A, B) Endogenous total and full‐length Parkin levels in wild‐type, *PRKN* ^c.100_101insC^, and *PINK* ^1‐KO SH SY5Y^ neuroblastoma cells following mitochondrial depolarization with 1 μM valinomycin across multiple time points complementary to main Figure 4. (A, B) Expression differences of total Parkin (full‐length Parkin + Parkin^Δ1–79^) between cell lines (B) and over increased durations of mitochondrial depolarization (B). Expression differences of only full‐length Parkin between cell lines (C) and over increased durations of mitochondrial depolarization (D). Sample size: n = 3 from independently repeated experiments across three cell passages. The significance threshold was set to *p* = 0.05. Whiskers extend to the largest and smallest values no further than 1.5*IQR from the hinge. Pairwise comparisons of linear mixed effects model derived estimated marginal means were Holm‐adjusted. (E–G) Assessment of Parkin translocation after 1 μM valinomycin‐induced mitochondrial depolarization by western blot analysis. Cytosolic and mitochondrial fractions were isolated from *PRKN* ^c.100_101insC^ (E), wild‐type (F), and *PINK* ^1‐KO^ (G) neuroblastoma cells (n = 1).
**Supplementary Figure S5.** Total Parkin (full‐length Parkin + Parkin^Δ1–79^) expression differences between groups (A) and expression changes with increased mitochondrial depolarization periods (B) in hiPSC‐derived midbrain dopaminergic neurons complementary to data in main Figure 5. Sample sizes represent a combination of individual patient‐derived neurons and independently repeated experiments across three differentiations. The significance threshold was set to *p* = 0.05. Whiskers extend to the largest and smallest values no further than 1.5*IQR from the hinge. Post hoc multiple comparisons via Durbin‐Conover tests (B) were Holm‐adjusted. (C) Relative Parkin mRNA expressed in iDNs. Whiskers show 1.5*IQR error propagated from technical replicates.
**Supplementary Figure S6.** Remaining endogenous MFN2 ubiquitination in biallelic *PRKN* ^delEx2^ patient fibroblasts. (A) Western blot analysis of MFN2 in 1 μM valinomycin‐treated fibroblasts of healthy controls (n = 6), a biallelic *PRKN* ^delEx2^ carrier (n = 6), and carriers of other *PRKN* variants downstream of the internal translation initiation site (n = 12). Membranes were reprobed without stripping to detect GAPDH as a loading control (lower panels). (B, C) Ubiquitination efficiency differences following mitochondrial depolarization between *PRKN* variant carriers and healthy controls (B) and change in ubiquitination efficiency between basal and depolarized states (C). Sample sizes represent a combination of individual fibroblast lines and independently repeated experiments across three to six passages. The significance threshold was set to *p* = 0.05. Whiskers extend to the largest and smallest values no further than 1.5*IQR from the hinge. Post hoc multiple comparisons via Games‐Howell tests (B) were Holm adjusted.

**Supplementary Data S1.** Supporting Information.

**Supplementary Data S2.** Supporting Information.

**Supplementary Data S3.** Supporting Information.

**Supplementary Data S4.** Supporting Information.

## Data Availability

Source data are provided with this study as part of the article and the supplementary data. All data presented and analyzed in this study are also available from the corresponding authors upon request.
