# Supplementary material for: Alternative Translation Initiation in PRKN Delays the Onset of Parkinson's Disease and Offers a Therapeutic Target
Source: Ann Neurol. 2026 Feb 22;99(6):1379–93. doi: 10.1002/ana.78180 (PMC13206481; doi:10.1002/ana.78180)
Supplement: Supplementary file 1 — Supplementary Figure S1. The hiPSC characterization. (C) Increased relative mRNA expression of the pluripotency markers GDF3. NANOG, OCT, and SOX2 in all newly generated and CRISPR‐Cas9 edited cell lines compared to Fibroblasts. (D) Increased relative mRNA expression of the ectodermal markers NCAM and PAX6, the mesodermal markers MSX1, MYH6, and RUNX1 as well as the endodermal markers GATA4 and SOX17 in embryoid bodies differentiated from all newly generated and CRISPR‐Cas9 edited cell lines compared to matched hiPSCs. (E) PCR amplification of PRKN Exon 1 to Exon 10 from iDN cDNA for all cell lines shown in main Figure 5. (F) Mycoplasma PCR test of all newly generated and CRISPR‐Cas9 edited cell lines. (G) PCR verifying the absence of Sendai virus reprogramming components for all newly generated and CRISPR‐Cas9 edited cell lines. Supplementary Figure S2. Differentiation scheme to generate midbrain dopaminergic neurons from patient‐derived iPSCs. Neuronal precursor cells (NPCs) were plated on PDL/LA coated cell culture plates on day 20, as indicated in red. The iDNs were kept in culture until day 120. Created with BioRender. Supplementary Figure S3. Total Parkin analysis between groups (A) and within groups (B) complementary to overexpression experiments of three PRKN constructs in a PRKN knockout neuroblastoma cell model in SH‐SY5Y cells shown in main Figure 3. Sample size: n = 3 from independently repeated experiments across three cell passages. The significance threshold was set to p = 0.05. Whiskers extend to the largest and smallest values no further than 1.5*IQR from the hinge. Pairwise comparisons of linear mixed effects model derived estimated marginal means were Holm‐adjusted. Supplementary Figure S4. (A, B) Endogenous total and full‐length Parkin levels in wild‐type, PRKN c.100_101insC, and PINK 1‐KO SH SY5Y neuroblastoma cells following mitochondrial depolarization with 1 μM valinomycin across multiple time points complementary to main Figure 4. (A, B) Exp [file ANA-99-1379-s001.pdf]

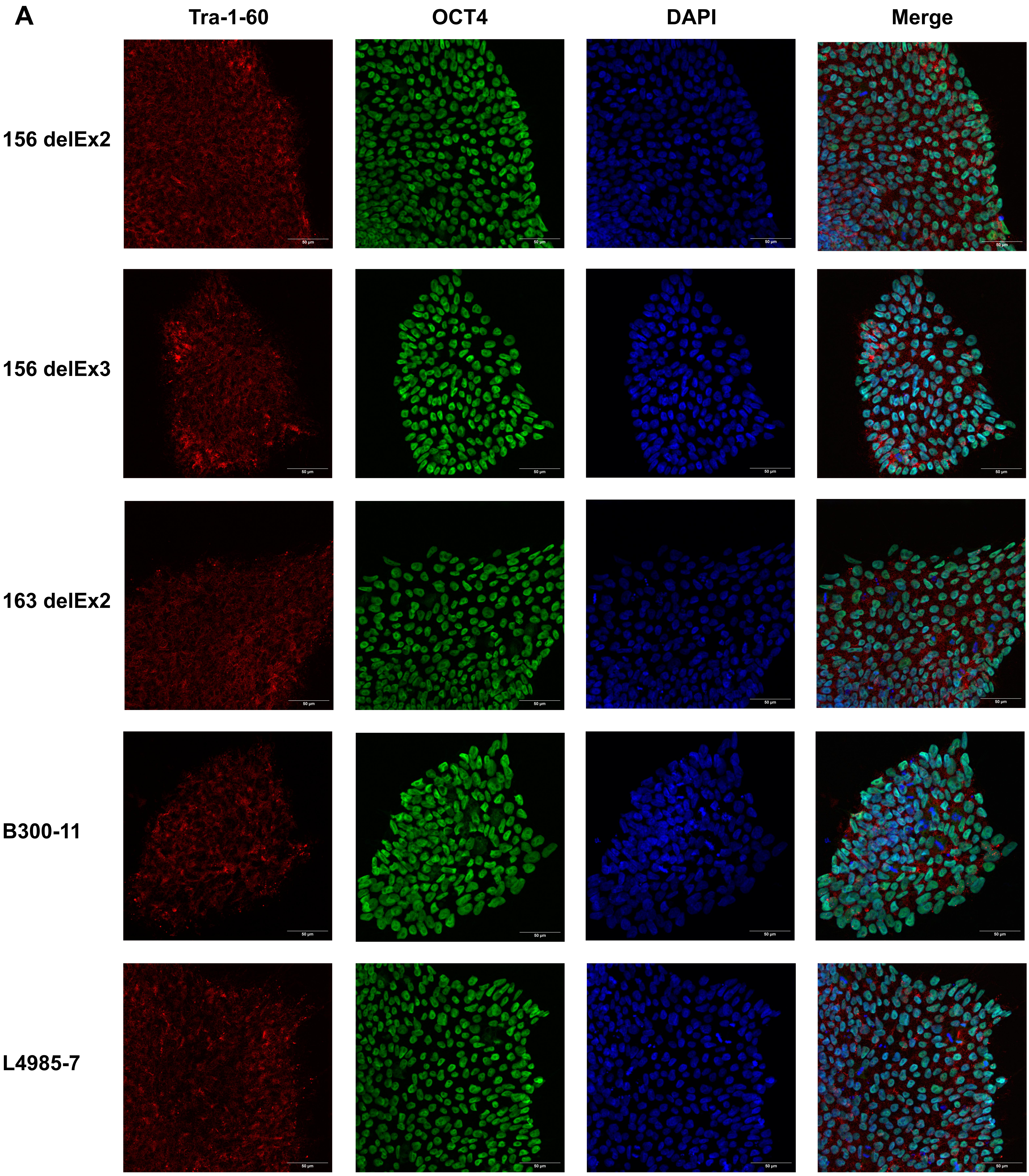

Supplementary Figure 1A: hiPSC characterization. Immunohistochemistry confocal microscopy images of all newly generated and CRISPR-Cas9 edited cell lines. Antibody staining for the pluripotency markers Tra-1-60 and OCT4. Nuclear counterstain via DAPI. Z-stacks were collapsed by average intensity projections.

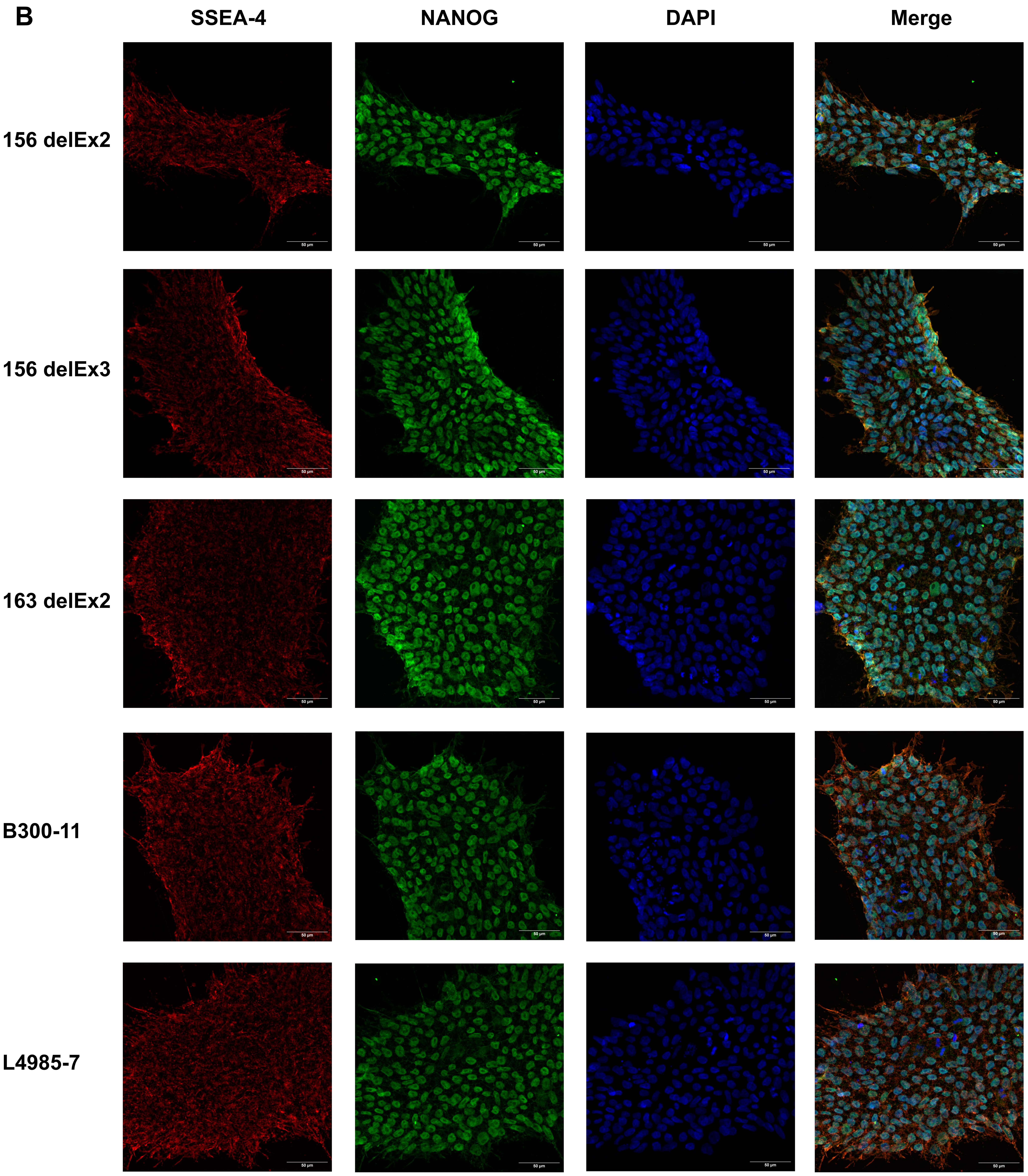

Supplementary Figure 1B: hiPSC characterization. Immunohistochemistry confocal microscopy images of all newly generated and CRISPR-Cas9 edited cell lines. Antibody staining for the pluripotency markers SSEA-4 and NANOG. Nuclear counterstain via DAPI. Z-stacks were collapsed by average intensity projections.

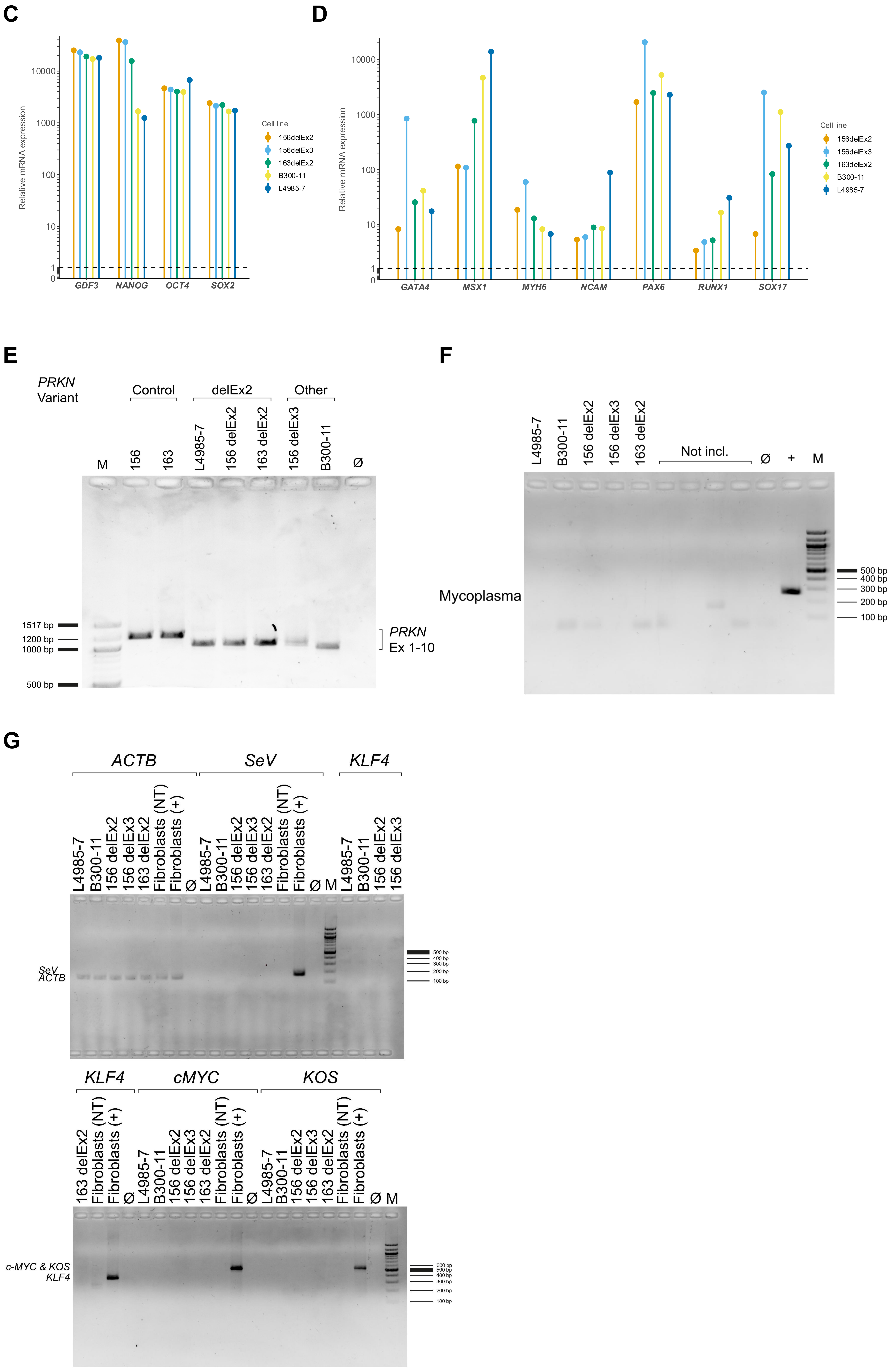

Supplementary Figure 1: hiPSC characterization. (**C**) Increased relative mRNA expression of the pluripotency markers *GDF3*. *NANOG*, *OCT*, and *SOX2* in all newly generated and CRISPR-Cas9 edited cell lines compared to Fibroblasts. (**D**) Increased relative mRNA expression of the ectodermal markers *NCAM* and *PAX6*, the mesodermal markers *MSX1*, *MYH6*, and *RUNX1* as well as the endodermal markers *GATA4* and *SOX17* in embryoid bodies differentiated from all newly generated and CRISPR-Cas9 edited cell lines compared to matched hiPSCs. (**E**) PCR amplification of *PRKN* Exon 1 to Exon 10 from iDN cDNA for all cell lines shown in main figure 5. **(F)** Mycoplasma PCR test of all newly generated and CRISPR-Cas9 edited cell lines. (**G**) PCR verifying the absence of Sendai virus reprogramming components for all newly generated and CRISPR-Cas9 edited cell lines.

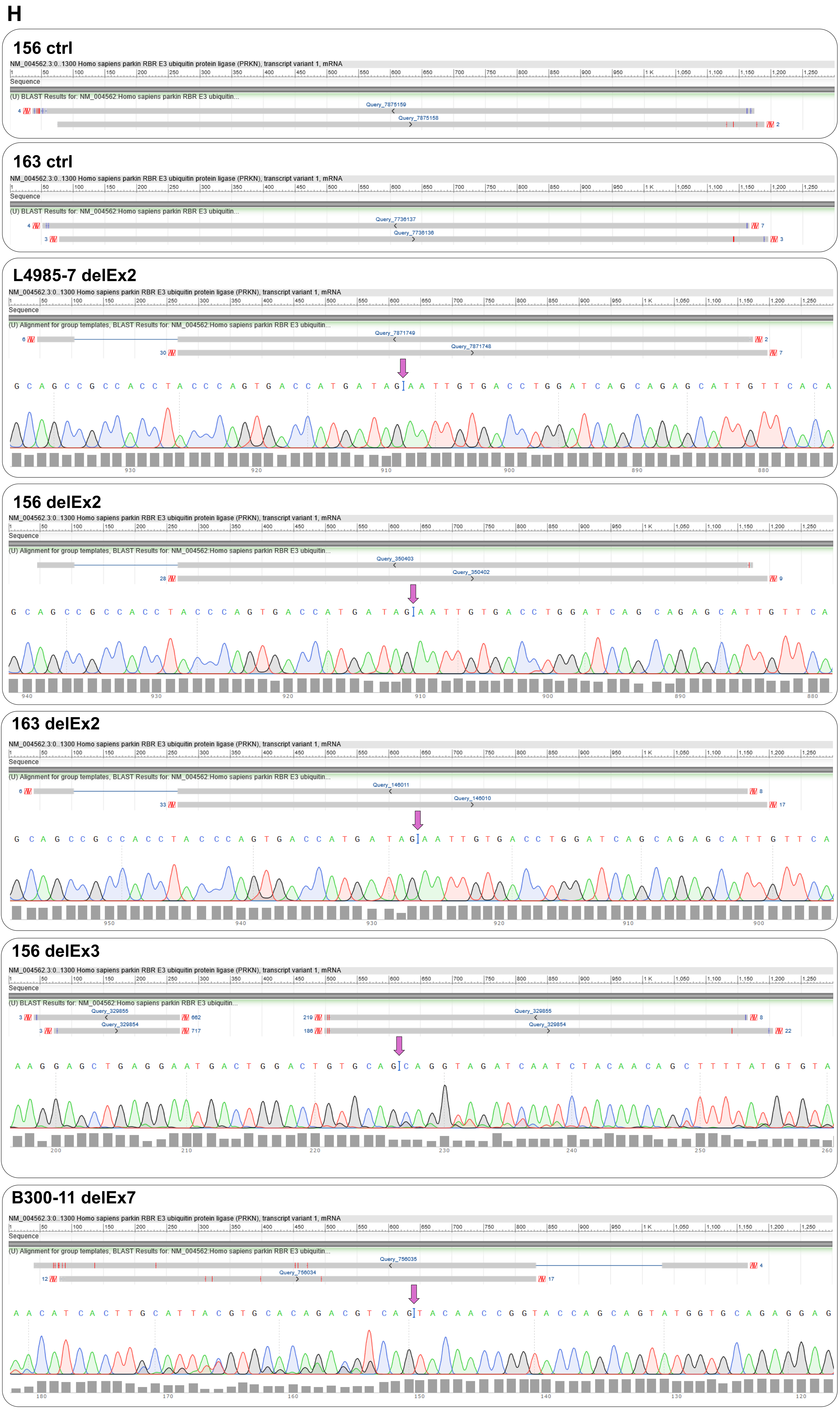

Supplementary Figure 1h: hiPSC characterization. Genotype confirmation by sanger sequencing using iDN cDNA amplified in (**E**). BLAST alignments to NM_004562.3 shown for all analyzed cell lines. Sequence windows of electropherograms relevant to each Exon deletion are shown. Arrows indicate the location of each variant. While patient B-300 is known to carry a homozygous deletion of Exon 7, disruption of the splicing context may result in the observed deletion of both Exon 7 & 8 on mRNA level.

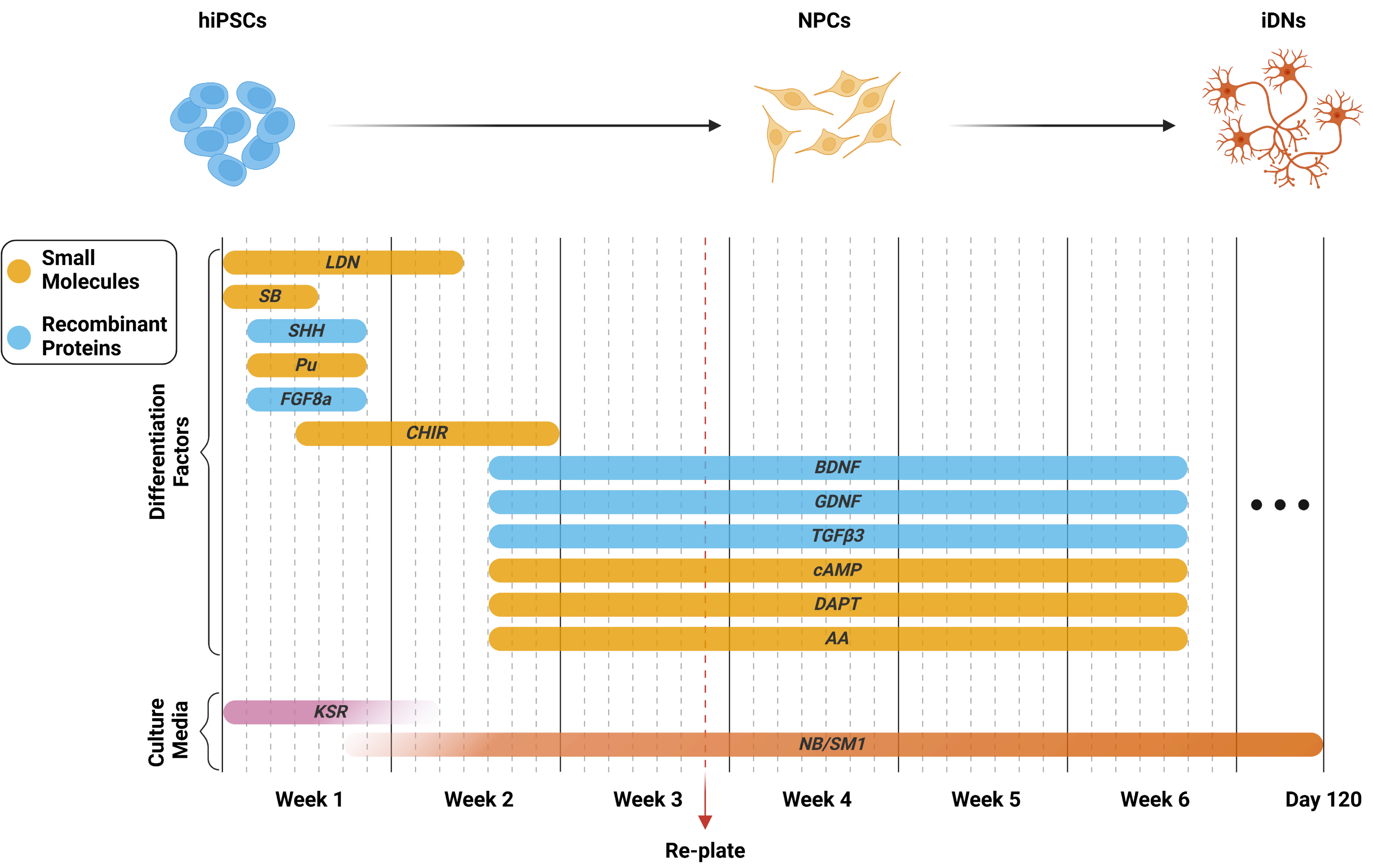

Supplementary Figure 2: Differentiation scheme to generate midbrain dopaminergic neurons from patient-derived iPSCs. Neuronal precursor cells (NPCs) were plated on PDL/LA coated cell culture plates on day 20, as indicated in red. iDNs were kept in culture until day 120. Created with BioRender.

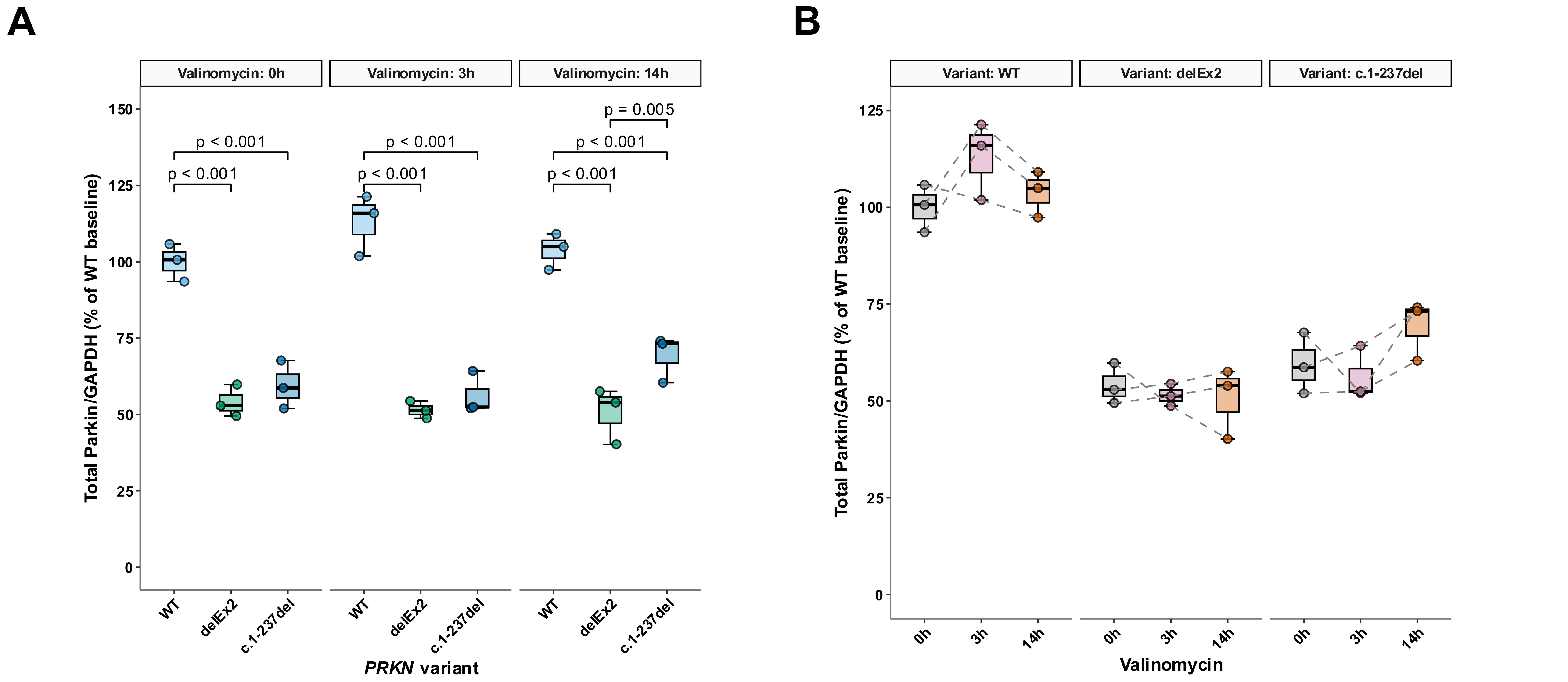

Supplementary Figure 3: Total Parkin analysis between groups (**A**) and within groups (**B**) complementary to overexpression experiments of three *PRKN* constructs in a *PRKN* knockout neuroblastoma cell model in SH-SY5Y cells shown in main figure 3. Sample size: n = 3 from independently repeated experiments across three cell passages. The significance threshold was set to p = 0.05. Whiskers extend to the largest and smallest values no further than 1.5 * IQR from the hinge. Pairwise comparisons of linear mixed effects model derived estimated marginal means were Holm-adjusted.

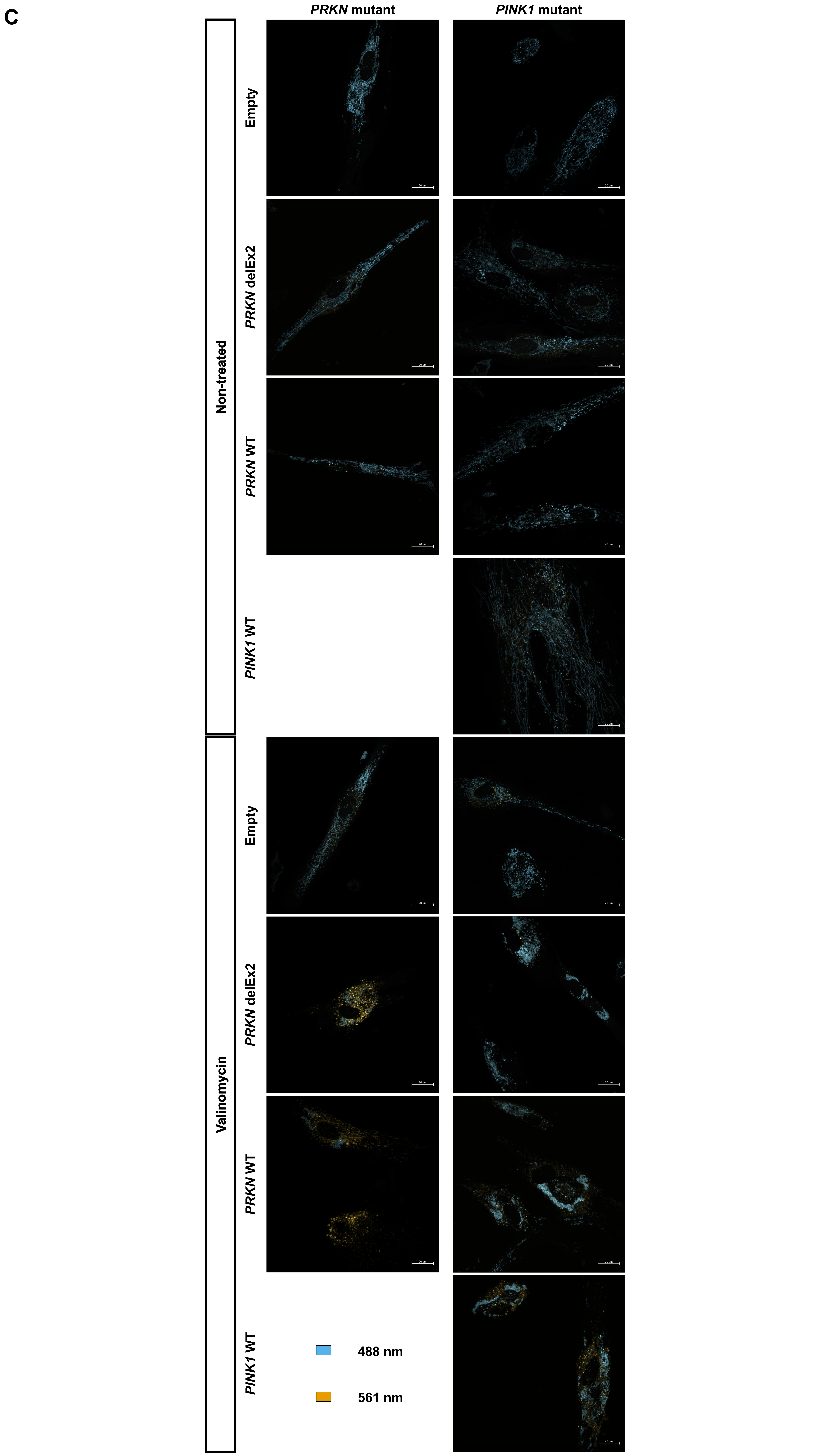

Supplementary Figure 3: (C) Confocal images corresponding to Fig. 3K, displayed using the full dynamic range of each channel.

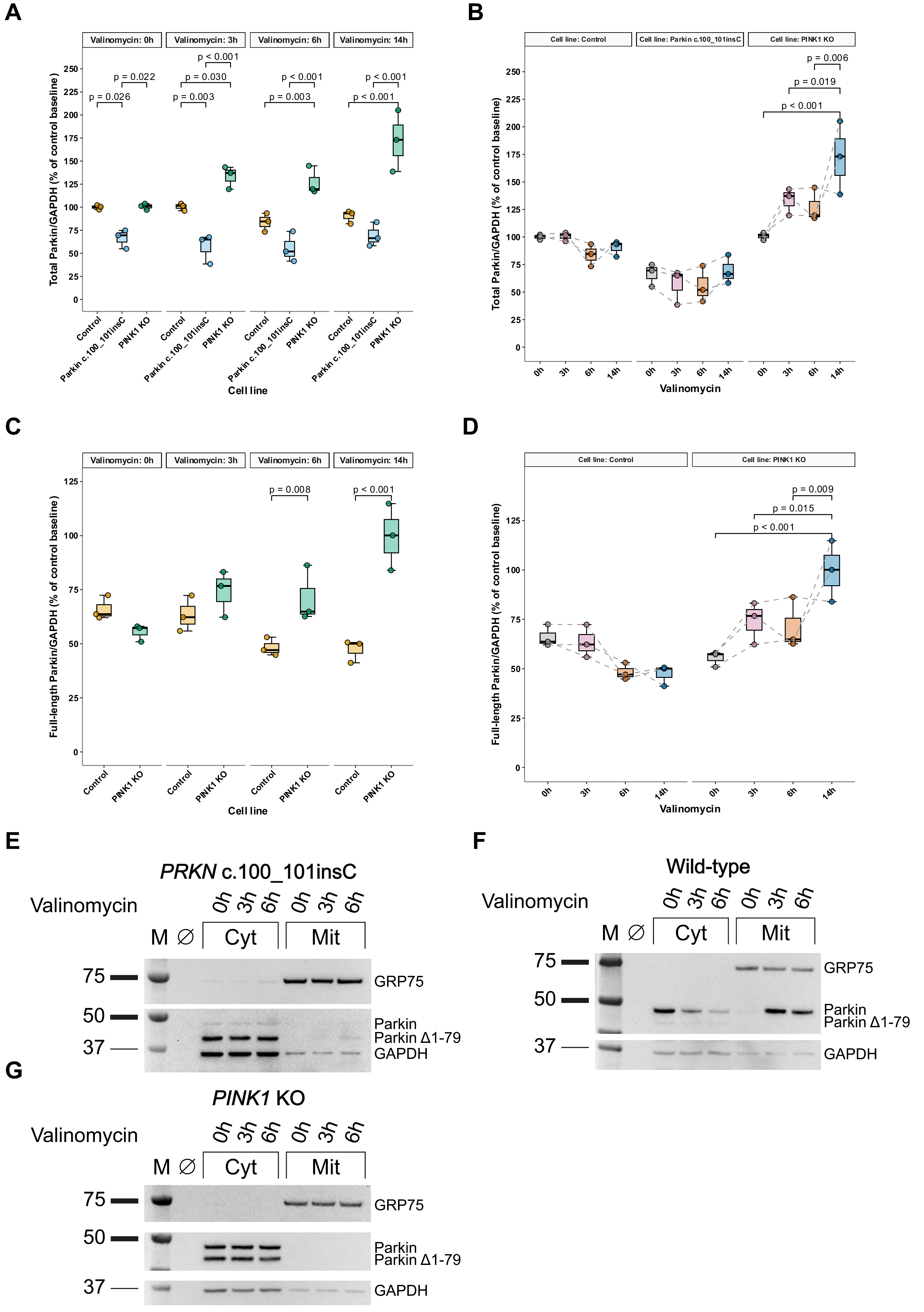

Supplementary Figure 4: (**A-B**) Endogenous total and full-length Parkin levels in wild-type, *PRKN*$^{c.100\_101insC}$, and *PINK1*-KO SH-SY5Y neuroblastoma cells following mitochondrial depolarization with 1 µM valinomycin across multiple time points complementary to main figure 4. (**A-B**) Expression differences of total Parkin (full-length Parkin + Parkin$^{\Delta 1-79}$) between cell lines (**B**) and over increased durations of mitochondrial depolarization (**B**). Expression differences of only full-length Parkin between cell lines (**C**) and over increased durations of mitochondrial depolarization (**D**). Sample size: n = 3 from independently repeated experiments across three cell passages. The significance threshold was set to p = 0.05. Whiskers extend to the largest and smallest values no further than 1.5 * IQR from the hinge. Pairwise comparisons of linear mixed effects model derived estimated marginal means were Holm-adjusted. (**E-G**) Assessment of Parkin translocation after 1 µM valinomycin-induced mitochondrial depolarization by western blot analysis. Cytosolic and mitochondrial fractions were isolated from *PRKN*$^{c.100_101insC}$ (**E**), wild-type (**F**), and *PINK1*-KO (**G**) neuroblastoma cells (n = 1).

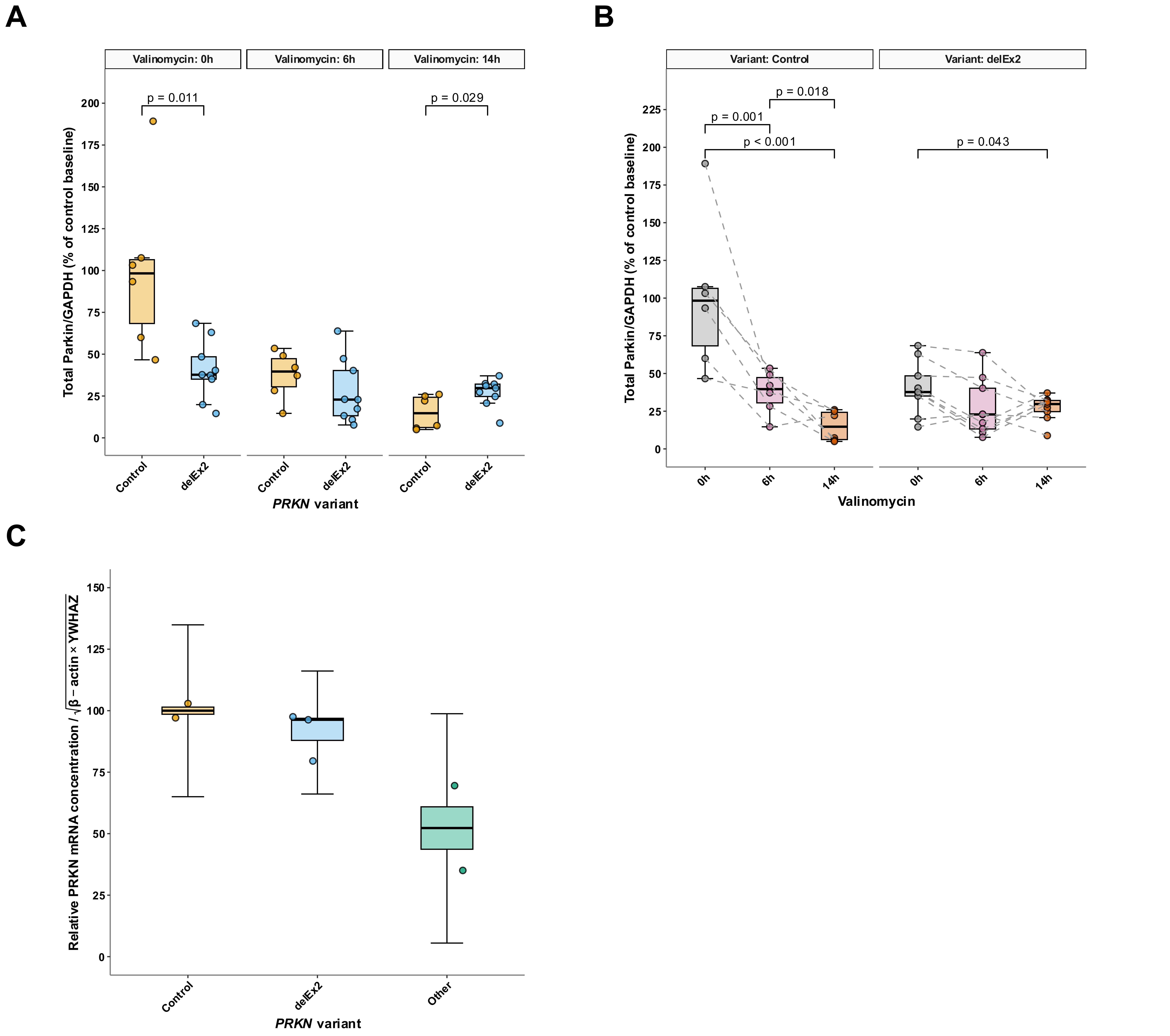

Supplementary Figure 5: Total Parkin (full-length Parkin + Parkin$^{\Delta 1-79}$) expression differences between groups (**A**) and expression changes with increased mitochondrial depolarization periods (**B**) in hiPSC-derived midbrain dopaminergic neurons complementary to data in main figure 5. Sample sizes represent a combination of individual patient-derived neurons and independently repeated experiments across three differentiations. The significance threshold was set to p = 0.05. Whiskers extend to the largest and smallest values no further than 1.5 * IQR from the hinge. Post-hoc multiple comparisons via Durbin-Conover tests (**B**) were Holm-adjusted. (**C**) Relative Parkin mRNA expressed in iDNs. Whiskers show 1.5 * IQR error propagated from technical replicates.

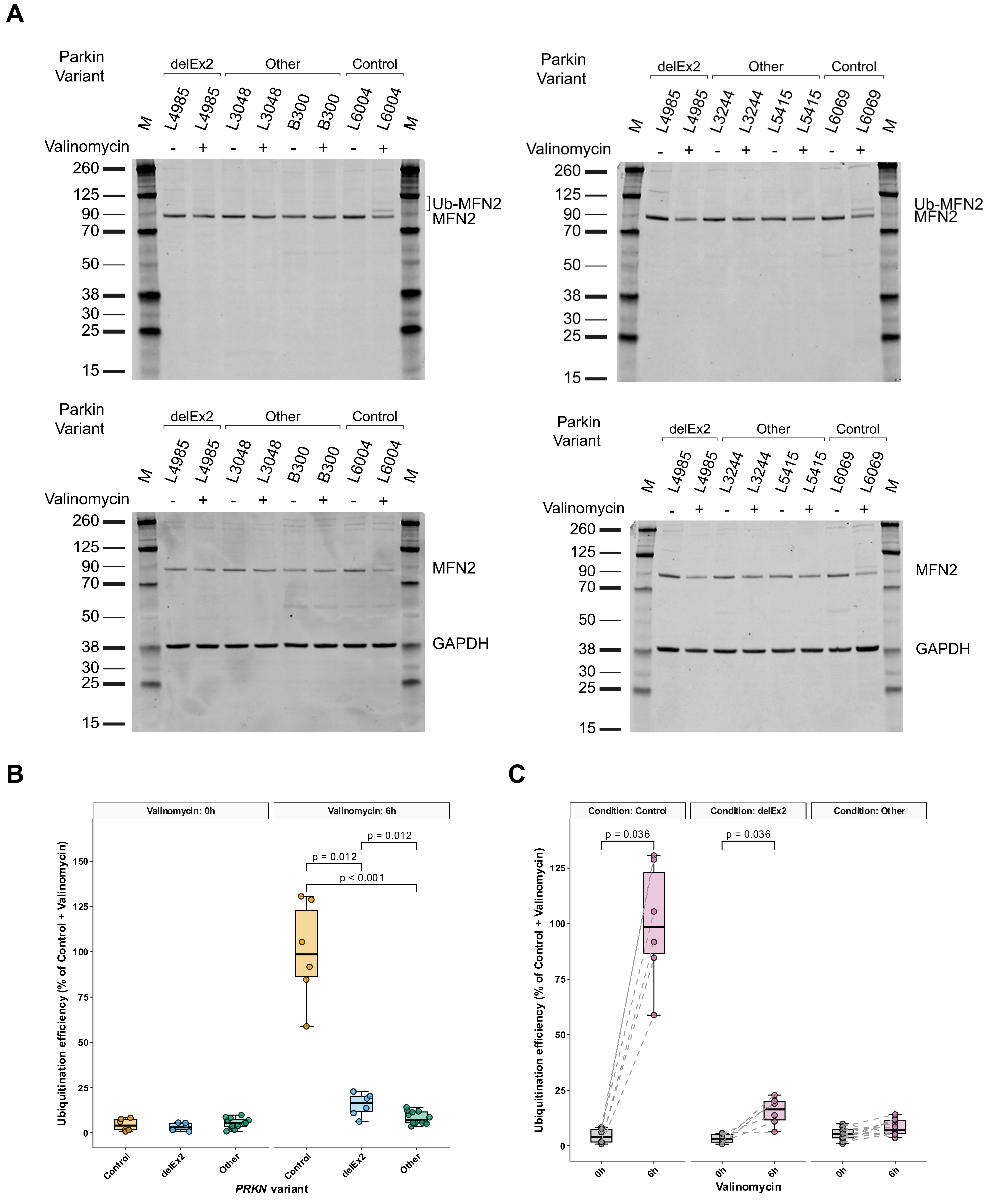

Supplementary Figure 6: Remaining endogenous MFN2 ubiquitination in biallelic *PRKN*^{delEx2} patient fibroblasts. (**A**) Western blot analysis of MFN2 in 1 µM valinomycin-treated fibroblasts of healthy controls (n = 6), a biallelic *PRKN*^{delEx2} carrier (n = 6), and carriers of other *PRKN* variants downstream of the internal translation initiation site (n = 12). Membranes were reprobed without stripping to detect GAPDH as a loading control (lower panels). (**B-C**) Ubiquitination efficiency differences following mitochondrial depolarization between *PRKN* variant carriers and healthy controls (**B**) and change in ubiquitination efficiency between basal and depolarized states (**C**). Sample sizes represent a combination of individual fibroblast lines and independently repeated experiments across three to six passages. The significance threshold was set to p = 0.05. Whiskers extend to the largest and smallest values no further than 1.5 * IQR from the hinge. Post-hoc multiple comparisons via Games-Howell tests (**B**) were Holm adjusted.
