# Supplementary material for: Alternative Translation Initiation in PRKN Delays the Onset of Parkinson's Disease and Offers a Therapeutic Target
Source: Ann Neurol. 2026 Feb 22;99(6):1379–93. doi: 10.1002/ana.78180 (PMC13206481; doi:10.1002/ana.78180)
Supplement: Supplementary file 2 — Supplementary Data S1. Supporting Information. [file ANA-99-1379-s005.docx]

## Supplementary Methods

### Dopaminergic midbrain neuron differentiation

Differentiation of characterized hiPSCs (Supplementary Fig. 1) into midbrain dopamine neurons was performed following an established protocol with minor alterations (Supplementary Fig. 2) ^1,2^. In brief, floorplate induction was initiated in hiPSCs by adding knockout serum replacement (KSR) medium supplemented with 10 µM SB431542 (Tocris) and 100nM LDN-193189 (Stemgent). 100 ng/ml recombinant human Sonic Hedgehog (rhSHH, STEMCELL), 100 ng/ml recombinant human fibroblast growth factor 8a (rhFGF-8a, STEMCELL), and 2 µM Purmorphamine from days 1-5. Furthermore, 3 µM CHIR99021 (STEMCELL) was added from days 3-13. SB431542 and LDN-193189 were withdrawn on days 5 and 11, respectively. KSR medium was gradually phased to Neurobasal medium (NB, Gibco) with NeuroCult SM1 neuronal supplement (STEMCELL) (25%, 50%, 75%, 100%) from days 5-10. From days 11-40, neural induction was started by adding 20 ng/ml brain-derived neurotrophic factor (BDNF, STEMCELL), 200 µM ascorbic acid (AA, Sigma), 20 ng/ml glial cell-derived neurotrophic factor (GDNF, STEMCELL), 1 ng/ml transforming growth factor-β3 (TGF-β3, PeproTech), 500 µM cyclic adenosine monophosphate (cAMP, Enzo), and 10 mM DAPT (Tocris). Cells were dissociated with Accutase (Thermo Fisher Scientific) on day 20 and replated on poly-D-lysine/Laminin/fibronectin-coated 6-well plates as high-density drops of 350,000 cells per 50 µL. From day 40, cells were maintained with NB/SM1. On day 120, dopaminergic midbrain neurons were treated with 1 µM valinomycin in NB/SM1 for 6 h and 14 h or left untreated.

To examine the effects of positive allosteric modulation on Parkin, neurons were pretreated with either 200 µM BIO-2007817 (Probechem) in DMSO (Sigma-Aldrich) or DMSO alone for 1 h, followed by 6 h of 1 µM valinomycin treatment. Cells were manually scraped and pelleted by centrifugation at 500 x *g* for 5 min on RT. Pellets were stored at -80 °C until further processing.

### Small molecule neuronal precursor cell differentiation

Differentiation of characterized hiPSCs into small-molecule neuronal precursor cells was performed using an established protocol.^3,4^ In brief, embryoid bodies (EBs) were generated from hiPSCs split via Accutase using AggreWell 400 plates (STEMCELL). For the first day, cells were kept in mTesR Plus (STEMCELL) supplemented with 10 µM Y-27632 (STEMCELL). On day 2, formed EBs were gently dislodged, filtered, and cultured on ultra-low attachment plates (Corning) in EB medium (KnockOut-DMEM (Gibco), 20% KnockOut-serum, 1% Penicillin/Streptomycin (Gibco), 1% GlutaMAX (Gibco), 1% Non-essential amino acids (Gibco), 100 µM 2-Mercaptoethanol (Gibco)) supplemented with three µM CHIR99021 (STEMCELL), 10 µM SB-431542 (Tocris), 1 µM Dorsomorphin (Tocris), and 0.5 µM Purmorphamine (Tocris). On day four, the medium was changed to N2B27 medium (48.25% DMEM/F12 (Gibco), 48,25% Neurobasal medium (Gibco), 1% Penicillin/Streptomycin, 1% GlutaMAX, 1% B-27 supplement without Vitamin A (Gibco), 0.5% N2 supplement (Gibco)) supplemented with the same concentration of small molecules. On day 6, the medium was changed to N2B27 supplemented with 3 µM CHIR99021, 0.75 µM Purmorphamine, and 150 µM ascorbic acid (Sigma). On day 8, EBs were split into single cells with Accutase and plated onto Matrigel (BD)-coated plates in N2B27 medium supplemented with the previous concentrations of small molecules and 10 µM Y-27632. The medium was replaced the following day with small-molecule-supplemented N2B27 medium without Y-27632. smNPCs were passaged every week.

### Induced pluripotent stem cell generation and characterization

The analyzed healthy control cell lines SFC156-03-01 (STBCi101-A) and SFC163-03-01 (STBCi102-A), which also served as parental lines for the isogenic *PRKN*^delEx2^ and *PRKN*^delEx3^ lines, have been reported previously.^5,6^ Fibroblast cultures from patient B-300 and the unaffected *PRKN*^delEx2^ carrier L-4985 were transduced to overexpress cMYC, KLF4, and KOS using the CytoTune-iPS 2.0 Sendai Reprogramming Kit (Thermo Fisher Scientific) according to the manufacturer’s instructions. The newly generated hiPSC and isogenic *PRKN*^delEx2^ and *PRKN*^delEx3^ cell lines were characterized to ensure correct genotype, pluripotency, clearance of Sendai virus components, and absence of mycoplasma contamination as previously described (Supplementary Fig. 1).^7,8^ In brief, embryoid bodies (EBs) were differentiated from each iPSC line by transferring cells to Ultra-Low Attachment 6-well plates (Corning) containing TeSR-E8-Medium containing 4mg/ml PVA (Sigma-Aldrich). After 2 days, the culture medium was changed to E6-Medium (Gibco). EBs were maintained in E6 for 14 days, changing the culture medium every other day. RNA was isolated from fresh iPSC and EB pellets (500 x g, 5 min) using the RNeasy Mini Kit (Qiagen). The RNA was reverse-transcribed using the First Strand cDNA Synthesis Kit (Thermo Fisher Scientific). PCR confirmed the absence of Sendai virus components and mycoplasma contamination. Here, fibroblasts 5 days after transduction and a mycoplasma-positive sample served as positive controls. To assess the expression of pluripotency markers *GDF3*, *NANOG*, *OCT4*, *SOX2* in hiPSCs and germ layer markers *GATA4*, *MSX1*, *MYH6*, *NCAM*, *PAX6*, *RUNX1*, and *SOX17* in EBs, quantitative real-time PCR runs were conducted with Maxima SYBR Green (Thermo Fisher Scientific) on the Lightcycler 96 (Roche). Additionally, iPSCs were fixed using 4% PFA and immunohistochemically stained for the pluripotency markers OCT4, NANOG, SSEA-4, and TRA-1-60. Furthermore, *PRKN* cDNA transcribed from iDNs was amplified from Exon 1(Primer F: GCGCGGCTGGCGCCGCTGGCGGCA) to Exon 10 (Primer R: GCTTCTTTACATTCCCGGCAG), run on an agarose gel, and analyzed by Sanger sequencing to confirm the *PRKN* genotype for each cell line. Additional primers to determine *PRKN* expression levels by quantitative real-time PCR were Exon 9F (TGACCAGAGGAAAGTCACCTG) and Exon 11R (CAGGGCTTGGTGGTTTTCTTG). All primers and antibodies not explicitly mentioned have been reported previously.^7,8^

### Viral vectors and transduction

Lentiviral expression plasmids for Parkin-wt, Parkin-delEx2, and Parkin-c.1-237del were generated by cloning cassettes containing the respective cDNA sequences, followed by an internal ribosomal entry site (IRES), and the puromycin N-acetyl-transferase (PAC) gene into NK57 lentiviral vectors.

Viral particles were produced in HEK 293FT cells by transfection with target, VSV-G, and 8.2ΔR plasmids using FuGENE HD (Promega) transfection reagent. Isogenic SH-SY5Y-wt and SH-SY5Y-PRKN-KO neuroblastoma cells were transduced with each lentiviral construct for 48h.

**Genome-edited cell lines**

We used several genome-edited neuroblastoma and hiPSC lines, including a previously generated isogenic SH-SY5Y cell line lacking PINK1 (SH-SY5Y *PINK1*^KO^).^9^ Further, SH-SY5Y *PRKN*^c.100_101insC^ cells, SH-SY5Y *PRKN*^KO^ (deletion of Exon 3), isogenic iPSC *PRKN*^delEx2^, and iPSC *PRKN*^delEx3^ cells were newly generated using an RNA-guided CRISPR/Cas9 endonuclease following previously established protocols.^10,11^ For the SH-SY5Y *PRKN*^c.100_101insC^ line, cells were transiently transfected with episomal vectors expressing both a human codon-optimized Cas9 and a guide RNA (gRNA) containing a 20-base-long sequence that matches the human *PRKN* target sequence 5’- GTGGTTGCTAAGCGACAGG-3’ in Exon 2. For iPSC *PRKN*^delEx2^, we used two gRNAs targeting 5’-TGTCAGGTTCAACTCCAGCC-3’ (in Exon 2) and 5’-GCAGGTGAGTCTCCCTTGG-3’ on the junction of Exon 2 and Intron 2-3. To generate SH-SY5Y *PRKN*^KO^ and iPSC *PRKN*^delEx3^, we used two gRNAs targeting 5’- TCAGCAGCTCAGTCCTCCC-3’ (in Exon 3) and 5’- AGCTGGAAGTCCAGGTAAT-3’ on the junction of Exon 3 and Intron 3-4. Upon transfection, cells were resuspended in the corresponding growth medium, counted, and plated onto Petri dishes at a density of 1 cell/cm2. Cells were grown until they formed distinct, monoclonal colonies. The colonies were scraped off, transferred into different wells of a 6-well plate, and propagated to obtain enough material for DNA extraction. All variants were confirmed by Sanger sequencing.

### Mitochondrial isolation

Fractionation of SH-SY5Y cells was conducted as previously described.^12,13^ Immediately after collection, cells were homogenized in an isolation medium containing 250 mM sucrose, 10 mM Tris-HCl, and 1 mM EDTA at pH 7.4. Homogenates were centrifuged for 20 min at 1,500 x g. Supernatants were transferred to a fresh tube and centrifuged for an additional 10 min at 12,000 x g. Supernatants containing cytosolic fractions were transferred to a fresh tube. Pellets containing the mitochondria were resuspended in Radioimmunoprecipitation assay (RIPA) buffer comprised of 25 mM Tris-HCl, 150 mM NaCl, 1% NP-40, 1% sodium deoxycholate, and 0.1% SDS at pH 7.6, supplemented with cOmplete protease and phosSTOP phosphatase inhibitors (Roche). Proteins in cytosolic fractions were concentrated with Amicon Ultracel 10K centrifugal filters (Millipore). Subsequently, fractions were analyzed by western blotting. Parkin^Δ1–79^ translocation was quantified via a translocation index defined as:

$$\frac{\left( \frac{\mathrm{Parkin}^{\Delta1-79}}{VDAC} \right)_{MIT}}{\left( \frac{\mathrm{Parkin}^{\Delta1-79}}{\beta-Tubulin} \right)_{CYT}}$$

### Antibodies

Primary antibodies raised against Parkin (Santa Cruz; sc32282; 1:1000), MFN2 (abcam; ab56889; 1:1000), GRP-75 (abcam; ab53098; 1:10^6^), TH (Millipore; AB152; 1:1000), and GAPDH (Cell Signaling; 14C10; 1:50000) were used. Parkin antibody dilution was adjusted for the detection of all overexpressed Parkin constructs (1:10^6^). HRP-conjugated goat anti-mouse (LI-COR; 926-80010; 1:20000) and goat anti-rabbit (LI-COR; 926-80011; 1:20000) were applied as secondary antibodies.

### mt-mKeima assay

Fibroblasts from three *PRKN*-PD patients (B300, L3048, L3244, Supplementary Table 2) and Fibroblasts from one *PINK1*-PD patient were transiently transfected with the pHAGE-mt-mKeima Plasmid (Addgene) in addition to an empty vector, and the lentiviral expression plasmids previously used in SH-SY5Y cells (Supplementary Data S1). *PINK1*-PD Fibroblasts were additionally transfected with an analogous plasmid expressing PINK1. Transfected cells and non-transfected controls were plated onto 8-well µSlides (ibidi) and selected with puromycin for 1-2 days until no cells remained in the non-transfected controls. Fibroblasts were treated with 1 µM valinomycin 12 h before confocal imaging. Live-cell imaging was performed using an LSM 900 confocal microscope and an Airyscan 2 module (Zeiss). Cells were imaged using the multiplex SR-4Y mode and a 40x objective (EC Plan-Neofluar 40x/1.30 Oil DIC M27) without averaging. All laser and detector settings were calibrated using non-treated cells transfected with a control plasmid and *PRKN*-PD cells transfected with wild-type PRKN after depolarization to ensure a linear range of the signal. These settings were kept constant across all images. mt-mKeima confocal images were analyzed using the colocalization suite of the Zen Black software (Zeiss) as previously described.^14^ The mitophagy index data were aggregated across all z-stacks for each cell and analyzed using custom R scripts, as described in the statistical analysis section.

### *In silico* *PRKN* translation initiation site prediction

cDNA sequences of wild-type (NM_004562.3), delEx2, c.100_101insC, and c.2T>C *PRKN* variants were analyzed using TIS Transformer (Version 1.0) in conjunction with the transcript-transformer Python package (Version 0.4).^15^ The minimum prediction threshold was set to 0.001 to track model prediction outputs for the internal TIS across all analyzed sequences.

### Western blot analysis

Western blots were background-corrected and quantified using Bio-Rad Image Lab (Version 6.1) with rolling disc background subtraction. To sensitively assess and normalize ubiquitination efficiency across western blots of *in vitro* models endogenously expressing Parkin via the ratio of ubiquitinated MFN2 (Ub-MFN2) over MFN2, the following formula was applied:

$$\frac{\left( \frac{Ub-MFN2}{MFN2} \right)_{Variant}}{\left( \frac{Ub-MFN2}{MFN2} \right)_{Control}}$$

Data was normalized by the sum of each signal per blot to compare signals without antibody-internal normalization across blots.^16^ To this end, loading schemes were kept consistent across independently conducted western blots for each experiment. All signals were normalized to GAPDH, or fraction-wise to β-Tubulin and VDAC protein levels.

### Statistical Analysis

Western blot data were fitted to linear mixed-effects models to examine group, treatment time, and interaction effects, including sample ID per independent experiment as a random effect. Models thus had the following general structure: “normalizedSignal ~ Group * TreatmentTime + (1 | ID:Experiment)” Computation of simple main effects and post-hoc pairwise comparisons using the Kenward-Roger method for degrees of freedom approximation was performed with the emmeans package^17^. Where linear mixed-effects models failed to meet model assumptions, non-parametric Kruskal-Wallis tests followed by post-hoc Dunn tests or Friedman tests followed by post-hoc Durbin-Conover tests were used as alternatives. Where the assumptions did not hold, and comparisons were limited to two comparisons, Mann-Whitney U tests, and Wilcoxon’s tests were applied. Parkin^Δ1–79^ translocation indices were compared using the following linear mixed-effects model corresponding to a paired t-test:

“TranslocationIndex ~ Treatment + (1 | Blot)”.

Mt-Keima mitophagy confocal data were analyzed using the glmmTMB R package, given the heteroscedasticity and non-Gaussian nature of the mitophagy index.^18^ To account for the hierarchical experimental design, the following structure model was chosen: “MitophagyIndex ~ Plasmid * Treatment * Genotype + (1 | Cellline / FileID)”. Including cell lines and image fields as nested random effects. Heteroscedasticity between experimental groups was modeled using a dispersion formula dependent on all fixed factor interactions: “dispformula = ~ Plasmid * Treatment * Genotype”. Due to the bounded, zero-inflated (in non-treated samples), and skewed distribution of the mitophagy index, the ordered beta family was chosen for modeling. With the non-normal distribution of the response variable, exact degrees of freedom are not defined for the statistical inference of simple main effects and post-hoc pairwise contrasts. P-values are therefore based on the asymptotic Wald Z-distribution.

To assess the association between homozygous Exon 2 deletions in *PRKN* and AAO, the data were first propensity score-matched using the covariates sex and country of origin with the MatchIt package^19^. Subsequently, linear regression models of varying complexities were fitted to the data. To avoid overfitting, the corrected Akaike information criterion (AICc) was computed for each model, selecting the following: “AAO ~ delEx2.hmz + Sex + Country”. The DHARMa package was utilized to diagnose potential model misspecifications and assumption violations^20^. The marginal hazard ratio and cumulative incidences were calculated using a Cox proportional hazards regression model with clustering by subclassification of the matched data. Cox regression analysis was performed using the survival and adjustedCurves packages^21,22^. Patients with missing reports on the outcome, predictor, or covariates were excluded from the analysis. All post-hoc tests were Holm corrected. All tests were two-tailed, and the alpha threshold probability was set to 0.05. Experimental data and statistical test details are documented in the supplementary materials (S2 and S3).

## Supplementary references

1. Kriks S, Shim J-W, Piao J, et al. Dopamine neurons derived from human ES cells efficiently engraft in animal models of Parkinson’s disease. Nature 2011;480(7378):547–551.

2. Zanon A, Kalvakuri S, Rakovic A, et al. SLP-2 interacts with Parkin in mitochondria and prevents mitochondrial dysfunction in Parkin-deficient human iPSC-derived neurons and Drosophila. Hum Mol Genet 2017;26(13):2412–2425.

3. Reinhardt P, Glatza M, Hemmer K, et al. Derivation and expansion using only small molecules of human neural progenitors for neurodegenerative disease modeling. PLoS One 2013;8(3):e59252.

4. Rakovic A, Voß D, Vulinovic F, et al. Electrophysiological Properties of Induced Pluripotent Stem Cell-Derived Midbrain Dopaminergic Neurons Correlate With Expression of Tyrosine Hydroxylase [Internet]. Frontiers in Cellular Neuroscience 2022;16[cited 2023 Sep 28 ] Available from: https://www.frontiersin.org/articles/10.3389/fncel.2022.817198

5. STBCi101-A · Cell Line · hPSCreg [Internet]. [date unknown];[cited 2025 Apr 11 ] Available from: https://hpscreg.eu/cell-line/STBCi101-A

6. STBCi102-A · Cell Line · hPSCreg [Internet]. [date unknown];[cited 2025 Apr 11 ] Available from: https://hpscreg.eu/cell-line/STBCi102-A

7. Baumann H, Jahn M, Muenchau A, et al. Generation and characterization of eight human-derived iPSC lines from affected and unaffected *THAP1* mutation carriers. Stem Cell Research 2018;33:60–64.

8. Tanzer K, Meier B, Vulinovic F, et al. Generation of four human-derived iPSC TorsinA-3xFLAG reporter lines from a DYT-TOR1A patient. Stem Cell Research 2024;81:103595.

9. Rakovic A, Ziegler J, Mårtensson CU, et al. PINK1-dependent mitophagy is driven by the UPS and can occur independently of LC3 conversion. Cell Death Differ 2019;26(8):1428–1441.

10. Rakovic A, Domingo A, Grütz K, et al. Genome editing in induced pluripotent stem cells rescues TAF1 levels in X-linked dystonia-parkinsonism. Mov Disord 2018;33(7):1108–1118.

11. Prasuhn J, Mårtensson CU, Krajka V, et al. Genome-Edited, TH-expressing Neuroblastoma Cells as a Disease Model for Dopamine-Related Disorders: A Proof-of-Concept Study on DJ-1-deficient Parkinsonism. Front Cell Neurosci 2017;11:426.

12. Almeida A, Medina JM. Isolation and characterization of tightly coupled mitochondria from neurons and astrocytes in primary culture. Brain Res 1997;764(1–2):167–172.

13. Rakovic A, Shurkewitsch K, Seibler P, et al. Phosphatase and Tensin Homolog (PTEN)-induced Putative Kinase 1 (PINK1)-dependent Ubiquitination of Endogenous Parkin Attenuates Mitophagy. J Biol Chem 2013;288(4):2223–2237.

14. Sun N, Malide D, Liu J, et al. A fluorescence-based imaging method to measure in vitro and in vivo mitophagy using mt-Keima. Nat Protoc 2017;12(8):1576–1587.

15. Clauwaert J, McVey Z, Gupta R, Menschaert G. TIS Transformer: remapping the human proteome using deep learning. NAR Genom Bioinform 2023;5(1):lqad021.

16. Degasperi A, Birtwistle MR, Volinsky N, et al. Evaluating Strategies to Normalise Biological Replicates of Western Blot Data. PLOS ONE 2014;9(1):e87293.

17. Lenth RV. emmeans: Estimated Marginal Means, aka Least-Squares Means [Internet]. 2025;Available from: https://rvlenth.github.io/emmeans/

18. Brooks ME, Kristensen K, Benthem KJ van, et al. glmmTMB Balances Speed and Flexibility Among Packages for Zero-inflated Generalized Linear Mixed Modeling. The R Journal 2017;9(2):378–400.

19. Ho D, Imai K, King G, Stuart EA. MatchIt: Nonparametric Preprocessing for Parametric Causal Inference. Journal of Statistical Software 2011;42:1–28.

20. Hartig F. DHARMa: Residual Diagnostics for Hierarchical (Multi-Level / Mixed) Regression Models. [Internet]. 2022;Available from: http://florianhartig.github.io/DHARMa/

21. Denz R, Klaaßen-Mielke R, Timmesfeld N. A comparison of different methods to adjust survival curves for confounders. Statistics in Medicine 2023;42(10):1461–1479.

22. Therneau TM. A Package for Survival Analysis in R [Internet]. 2024;Available from: https://CRAN.R-project.org/package=survival

23. Uchihara Y, Kataoka H, Yoshino H, et al. Parkin mutation may be associated with serious akinesia in a patient with Parkinson’s disease. J Neurol Sci 2017;379:119–121.

24. Kobayashi T, Wang M, Hattori N, et al. Exonic deletion mutations of the Parkin gene among sporadic patients with Parkinson’s disease. Parkinsonism Relat Disord 2000;6(3):129–131.

25. Kobayashi T, Matsumine H, Zhang J, et al. Pseudo-autosomal dominant inheritance of PARK2: two families with parkin gene mutations. J Neurol Sci 2003;207(1–2):11–17.

26. Kunishige M, Mitsui T, Kuroda Y, et al. Expanding phenotype and clinical heterogeneity in patients with identical mutation of the parkin gene. Eur Neurol 2004;51(3):183–185.

27. Zhang B, Hu Z, Yin X, et al. Mutation analysis of parkin and PINK1 genes in early-onset Parkinson’s disease in China. Neurosci Lett 2010;477(1):19–22.

28. Maruyama M, Ikeuchi T, Saito M, et al. Novel mutations, pseudo-dominant inheritance, and possible familial affects in patients with autosomal recessive juvenile parkinsonism. Ann Neurol 2000;48(2):245–250.

29. Kitada T, Asakawa S, Hattori N, et al. Mutations in the parkin gene cause autosomal recessive juvenile parkinsonism. Nature 1998;392(6676):605–608.

30. Dogu O, Johnson J, Hernandez D, et al. A consanguineous Turkish family with early-onset Parkinson’s disease and an exon 4 parkin deletion. Mov Disord 2004;19(7):812–816.

31. Yoritaka A, Shimo Y, Shimo Y, et al. Nonmotor Symptoms in Patients with PARK2 Mutations. Parkinsons Dis 2011;2011:473640.

32. Hanagasi HA, Serdaroglu P, Ozansoy M, et al. Mitochondrial pathology in muscle of a patient with a novel parkin mutation. Int J Neurosci 2009;119(10):1572–1583.

33. Funayama M, Li Y, Tsoi T-H, et al. Familial Parkinsonism with digenic parkin and PINK1 mutations. Mov Disord 2008;23(10):1461–1465.

34. Infante J, Berciano J, Sánchez-Juan P, et al. Pseudo-orthostatic and resting leg tremor in a large Spanish family with homozygous truncating parkin mutation. Mov Disord 2009;24(1):144–147.

35. Muñoz E, Tolosa E, Pastor P, et al. Relative high frequency of the c.255delA parkin gene mutation in Spanish patients with autosomal recessive parkinsonism. J Neurol Neurosurg Psychiatry 2002;73(5):582–584.

36. Kumru H, Santamaria J, Tolosa E, et al. Rapid eye movement sleep behavior disorder in parkinsonism with parkin mutations. Ann Neurol 2004;56(4):599–603.

37. Shi Y, Kawakami H, Zang W, et al. Novel compound heterozygous mutations in the PARK2 gene identified in a Chinese pedigree with early-onset Parkinson’s disease. Brain Behav 2018;8(1):e00901.

38. Chen H, Huang X, Yuan L, et al. A homozygous parkin p.G284R mutation in a Chinese family with autosomal recessive juvenile parkinsonism. Neurosci Lett 2016;624:100–104.

39. Fang Y-Q, Mao F, Zhu M-J, Li X-H. Compound heterozygous mutations in PARK2 causing early-onset Parkinson disease: A case report. Medicine (Baltimore) 2019;98(5):e14228.

40. Lesage S, Lohmann E, Tison F, et al. Rare heterozygous parkin variants in French early-onset Parkinson disease patients and controls. J Med Genet 2008;45(1):43–46.

41. Periquet M, Latouche M, Lohmann E, et al. Parkin mutations are frequent in patients with isolated early-onset parkinsonism. Brain 2003;126(Pt 6):1271–1278.

42. Guo J, Zhang X, Nie L, et al. Mutation analysis of Parkin, PINK1 and DJ-1 genes in Chinese patients with sporadic early onset parkinsonism. J Neurol 2010;257(7):1170–1175.

43. Wu R-M, Bounds R, Lincoln S, et al. Parkin mutations and early-onset parkinsonism in a Taiwanese cohort. Arch Neurol 2005;62(1):82–87.

44. Koentjoro B, Park J-S, Ha AD, Sue CM. Phenotypic variability of parkin mutations in single kindred. Mov Disord 2012;27(10):1299–1303.

45. Bravo P, Darvish H, Tafakhori A, et al. Molecular characterization of PRKN structural variations identified through whole-genome sequencing. Mol Genet Genomic Med 2018;6(6):1243–1248.

46. Hedrich K, Kann M, Lanthaler AJ, et al. The importance of gene dosage studies: mutational analysis of the parkin gene in early-onset parkinsonism. Hum Mol Genet 2001;10(16):1649–1656.

47. Keyser RJ, Lombard D, Veikondis R, et al. Analysis of exon dosage using MLPA in South African Parkinson’s disease patients. Neurogenetics 2010;11(3):305–312.

48. De Rosa A, Pellegrino T, Pappatà S, et al. Myocardial 123I-metaiodobenzylguanidine scintigraphy in patients with homozygous and heterozygous parkin mutations. J Nucl Cardiol 2017;24(1):103–107.

49. Kim TJ, Kim TJ, Lee H, et al. A case of Parkin disease (PARK2) with schizophrenia: Evidence of regional selectivity. Clin Neurol Neurosurg 2014;126:35–37.

50. Jankovic MZ, Dobricic V, Kresojevic N, et al. Identification of mutations in the PARK2 gene in Serbian patients with Parkinson’s disease. J Neurol Sci 2018;393:27–30.

51. Thobois S, Ribeiro M-J, Lohmann E, et al. Young-onset Parkinson disease with and without parkin gene mutations: a fluorodopa F 18 positron emission tomography study. Arch Neurol 2003;60(5):713–718.

52. Chu MK, Kim WC, Choi JM, et al. Analysis of Dosage Mutation in PARK2 among Korean Patients with Early-Onset or Familial Parkinson’s Disease. J Clin Neurol 2014;10(3):244–248.

## Supplementary Tables

**Supplementary Table 1 Demographics of variant carriers reported on MDSGene and included in the AAO analysis propensity score matched to respective homozygous *PRKN*^delEx2^ variant carriers in Table 1**

| ID | Center | Country | Sex | AAO Range | AAE Range | Previously Reported | 1^st^ Variant | 2^nd^ Variant | Match Subclass | |
| --- | --- | --- | --- | --- | --- | --- | --- | --- | --- | --- |
| 1i | Nara Medical U | Japan | Male | 33 | 42 | Yes^23^ | delEx2 | delEx4 | 8 |  |
| 1i | Juntendo U | Japan | Male | 47 | 60 | Yes^24^ | delEx3 | delEx3 | 15 |  |
| II-2 | Juntendo U | Japan | Male | 22 | 45 | Yes^25^ | delEx3 | delEx3 | 12 |  |
| I-1 | Juntendo U | Japan | Male | 18 | NA | Yes^25^ | delEx3 | delEx3 | 11 |  |
| patient 2 | Tokushima U | Japan | Female | 19 | 59 | Yes^26^ | delEx3-4 | delEx3-4 | 14 |  |
| M5 | Zhejiang U | China | Female | 16 | 29 | Yes^27^ | delEx3-4 | delEx3-4 | 20 |  |
| IV-4 | Niigata U | Japan | Female | 19 | 35 | Yes^28^ | delEx4 | delEx4 | 16 |  |
| II-1 | Juntendo U | Japan | Male | 18 | NA | Yes^29^ | delEx4 | delEx4 | 10 |  |
| 479-10 | Mersin U | Turkey | Male | 16 | 32 | Yes^30^ | delEx4 | delEx4 | 26 |  |
| IV-3 | Niigata U | Japan | Male | 15 | 38 | Yes^28^ | delEx4 | delEx4 | 6 |  |
| IV-6 | Niigata U | Japan | Female | 8 | 30 | Yes^28^ | delEx4 | delEx4 | 9 |  |
| II-1 | Niigata U | Japan | Male | 30 | 35 | Yes^28^ | delEx6-7 | delEx6-7 | 5 |  |
| 13 | Juntendo U | Japan | Male | 45 | 57 | Yes^31^ | delEx2-4 | delEx2-4 | 25 |  |
| III-1 | Istanbul U | Turkey | Male | 37 | 53 | Yes^32^ | c.1084-1delG | c.1084-1delG | 17 |  |
| C2 | Juntendo U | China | Female | 18 | 35 | Yes^33^ | c.1321T>C | p.Ala138Glyfs*7 | 23 |  |
| III:1 | Cantabria U | Spain | Male | 30 | NA | Yes^34^ | c.1334G>A | c.1334G>A | 4 |  |
| PK-21 | Barcelona U | Spain | Male | 41 | 53 | Yes^35^ | c.155delA | c.155delA | 1 |  |
| 8 | IDIBAPS | Spain | Male | 39 | 56 | Yes^36^ | c.155delA | c.155delA | 2 |  |
| II-2 | HPPH | China | Female | 24 | 59 | Yes^37^ | c.619-1G>C | delEx1-2 | 19 |  |
| II-7 | HPPH | China | Male | 25 | NA | Yes^37^ | c.619-1G>C | delEx1-2 | 22 |  |
| II:1 | Central South U | China | Female | 22 | 29 | Yes^38^ | c.850G>C | c.850G>C | 24 |  |
| II:3 | Central South U | China | Male | 8 | 24 | Yes^38^ | c.850G>C | c.850G>C | 18 |  |
| II:1 | Shandong U | China | Male | 26 | 36 | Yes^39^ | c.850G>C | delEx6 | 21 |  |
| M6 | Zhejiang U | China | Male | 22 | 38 | Yes^27^ | delEx2 | delEx6 | 3 |  |
| A2 | Juntendo U | Japan | Male | 15 | 53 | Yes^33^ | p.Thr175Profs*2 | p.Thr175Profs*2 | 7 |  |
| A3 | Juntendo U | Japan | Female | 12 | 37 | Yes^33^ | p.Thr175Profs*2 | p.Thr175Profs*2 | 13 |  |

AAE = age at examination; U = University; IDIBAPS = Institut d’Investigacions Biomèdiques August Pi i Sunyer; HPPH = Henan Provincial People's Hospital.

**Supplementary Table 2 Human dermal fibroblasts analyzed in Fig. 3 and Supplementary Fig. 6**

| ID | AAE | Sex | Affected | Variant | Zygosity | Pathogenicity |
| --- | --- | --- | --- | --- | --- | --- |
| L4985 | 66 | Female | No | *PRKN* delEx2 | Hom | Definitely pathogenic |
| B300 | 45 | Female | Yes | *PRKN* delEx7 | Hom | Definitely pathogenic |
| L3048 | 57 | Male | Yes | *PRKN* delEx4; c.823C>T | Compound het | Definitely pathogenic |
| L5415 | 35 | Female | Yes | *PRKN* c.823C>T; c.1054T>C | Compound het | Definitely pathogenic |
| L3244 | 41 | Female | Yes | *PRKN* delEx1; c.823C>T | Compound het | Definitely pathogenic |
| L1703 | 73 | Female | Yes | *PINK1* c.509T>G | homozygous | Definitely pathogenic |
| L6069 | 74 | Male | No | WT | NA | NA |
| L6004 | 64 | Female | No | WT | NA | NA |

AAE = age at examination

**Supplementary Table 3 iPSC lines differentiated into midbrain dopaminergic neurons (iDN)**

| ID | AAE Range | Sex | Affected | *PRKN* variant | Zygosity | | Pathogenicity | Isogenic |
| --- | --- | --- | --- | --- | --- | --- | --- | --- |
| L4985-7 | 66 | Female | No | delEx2 | | Hom | Definitely pathogenic | NA |
| B300-11 | 45 | Female | Yes | delEx7 | | Hom | Definitely pathogenic | NA |
| SFC156-03-01 | 74 | Male | No | WT | | NA | NA | Parental |
| SFC156-03-01delEx2 | 74 | Male | NA | delEx2 | | Hom | Definitely pathogenic | Yes |
| SFC156-03-01delEx3 | 74 | Male | NA | delEx3 | | Hom | Definitely pathogenic | Yes |
| SFC163-03-01 | 62 | Male | No | WT | | NA | NA | Parental |
| SFC163-03-01delEx2 | 62 | Male | NA | delEx2 | | Hom | Definitely pathogenic | Yes |

AAE = age at examination

**Supplementary Table 4 Demographics of patients with a heterozygous *PRKN*^delEx2^ variant for *PRKN* (NM_004562.3) reported on MDSGene and included in the AAO analysis**

| ID | Country | Sex | AAO Range | AAE Range | Previously Reported | 1^st^ Variant | 2^nd^ Variant |
| --- | --- | --- | --- | --- | --- | --- | --- |
| SPD-322-010 | France | Male | 18 | 55 | Yes^40^ | delEx2 | 202_203delAG |
| SPD-169-003 | France | Male | 29 | 42 | Yes^41^ | delEx2 | delEx3 |
| SPD-166-010 | France | Male | 45 | 55 | Yes^41^ | delEx2 | delEx3-4 |
| Patient 4 | China | Female | 28 | 32 | Yes^42^ | delEx2 | 202_203delAG |
| M6 | China | Male | 22 | 38 | Yes^27^ | delEx2 | delEx6 |
| M335 | China | Female | 29 | 44 | Yes^27^ | delEx2 | delEx7-9 |
| II:3 | China | Male | 36 | 41 | Yes^43^ | delEx2 | c.951G>C |
| II:2 | China | Male | 32 | 44 | Yes^43^ | delEx2 | c.951G>C |
| II-a | Greece | Female | 18 | NA | Yes^44^ | delEx2 | delEx5-7 |
| FB_P1 | Iran | Female | 10 | 24 | Yes^45^ | delEx2 | delEx3 |
| B-151 | Italy | Male | 18 | 34 | Yes^46^ | delEx2 | delEx5 |
| 37.12 | South Africa | Male | 45 | NA | Yes^47^ | delEx2 | delEx9 |
| 3 | Italy | Male | 35 | 67 | Yes^48^ | delEx2 | delEx2-3 |
| 2 | Italy | Male | 22 | 63 | Yes^48^ | delEx2 | delEx2-3 |
| 1i | South Korea | Male | 10 | 20 | Yes^49^ | delEx2 | delEx4 |
| 1i | Japan | Male | 33 | 42 | Yes^23^ | delEx2 | delEx4 |
| 1i | Serbia | Female | 18 | NA | Yes^50^ | delEx2 | delEx3 |
| 19 | France | Male | 13 | 49 | Yes^51^ | delEx2 | delEx3 |
| 18 | France | Female | 40 | 46 | Yes^51^ | delEx2 | delEx3 |
| 17i | South Korea | Male | 28 | 34 | Yes^52^ | delEx2 | delEx4 |

AAE = age at examination
