# Supplementary material for: Alternative Translation Initiation in PRKN Delays the Onset of Parkinson's Disease and Offers a Therapeutic Target
Source: Ann Neurol. 2026 Feb 22;99(6):1379–93. doi: 10.1002/ana.78180 (PMC13206481; doi:10.1002/ana.78180)
Supplement: Supplementary file 5 — Supplementary Data S4. Supporting Information. [file ANA-99-1379-s004.pdf]

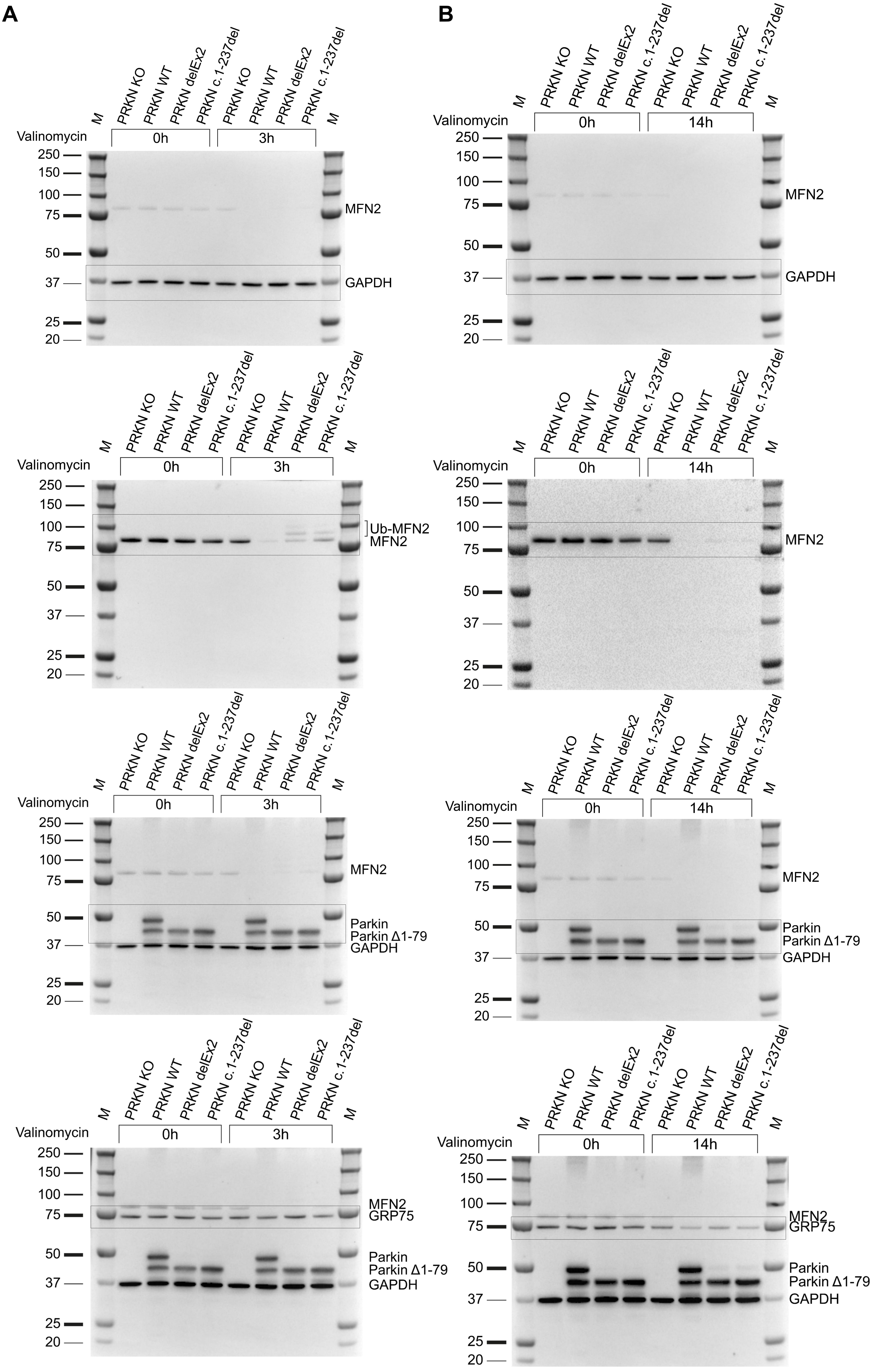

Complete western blots. Full blots after 3 h (**A**) and 14 h (**B**) valinomycin treatment in SH-SY5Y cells overexpressing Parkin as shown in main figure 3A, B. Blots are shown for each cropped signal, marked by boxes.

C

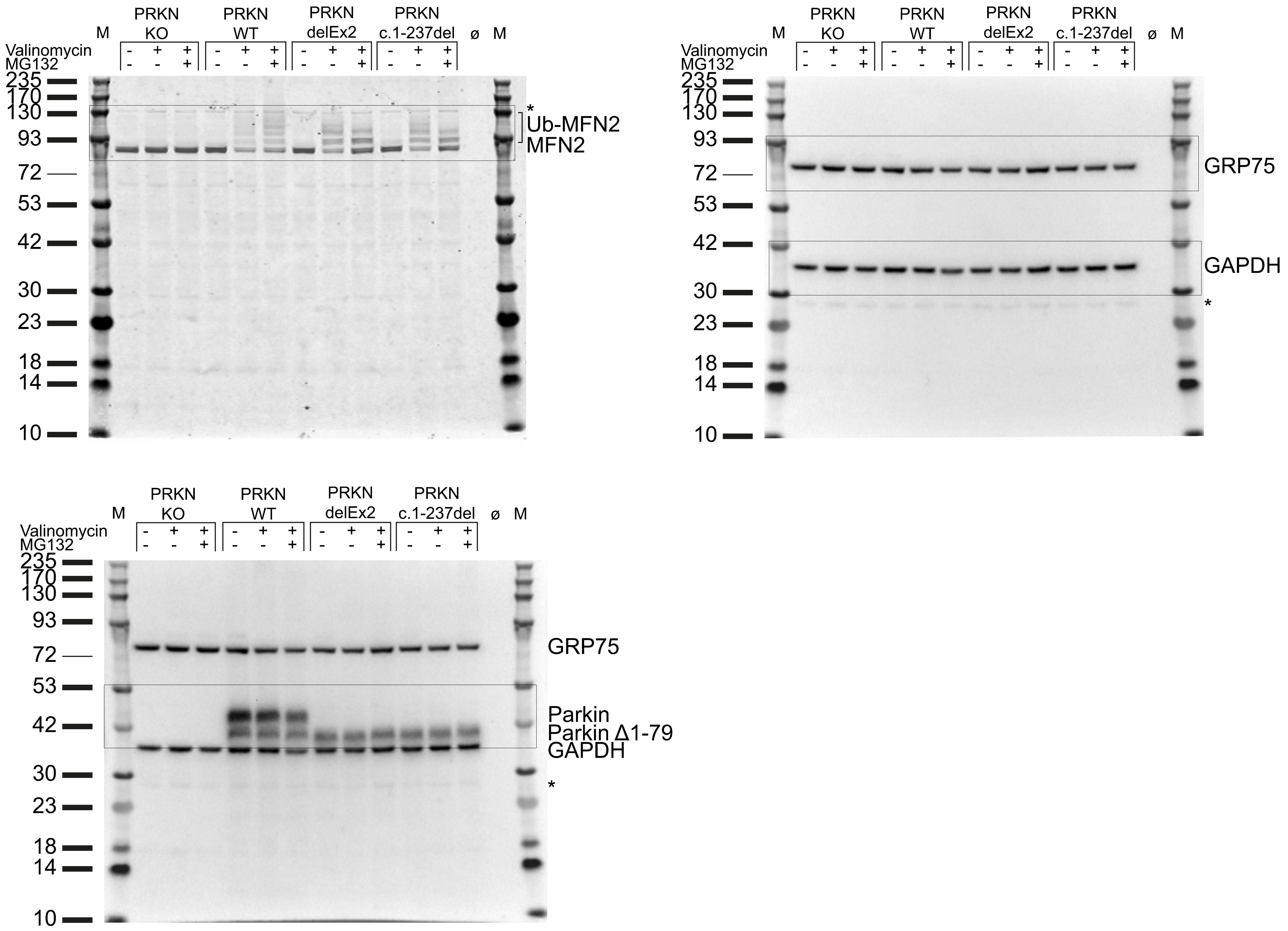

Complete western blots. (**C**) Full blots after 3 h valinomycin and MG132 treatment in SH-SY5Y cells overexpressing Parkin as shown in main figure 3C. Blots are shown for each cropped signal, marked by boxes.

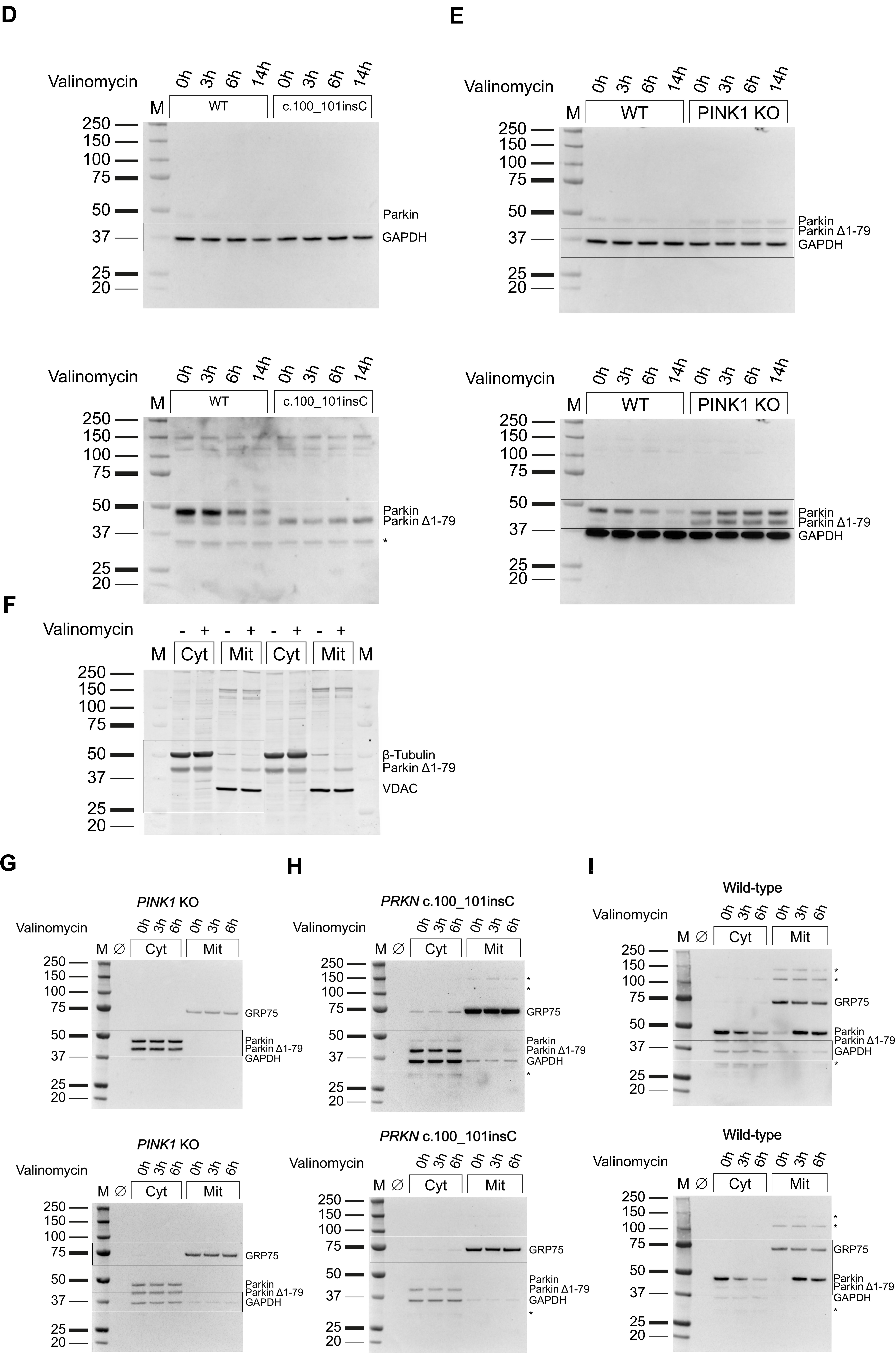

Complete western blots. Full blots for c.100_101insC and wildtype (**D**) or PINK1 KO and wildtype cells (**E**) for quantification of endogenous Parkin in SH-SY5Y cells as well as full blots for the fractionation analyses in smNPCs (**F**). Technical replicates, collapsed for the analysis were loaded in each half of (**F**). PINK1 KO (**G**) c.100_101inC (**H**) and wildtype (**I**) as shown in main figure 4 and supplementary figure 4. Blots are shown for each cropped signal, marked by boxes.

J

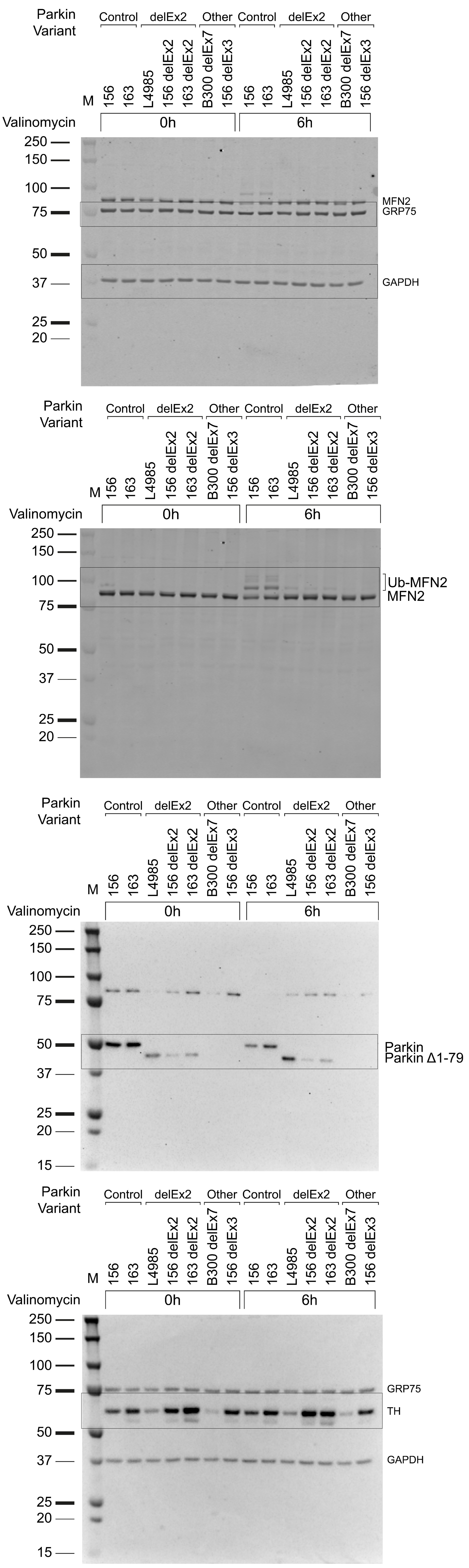

K

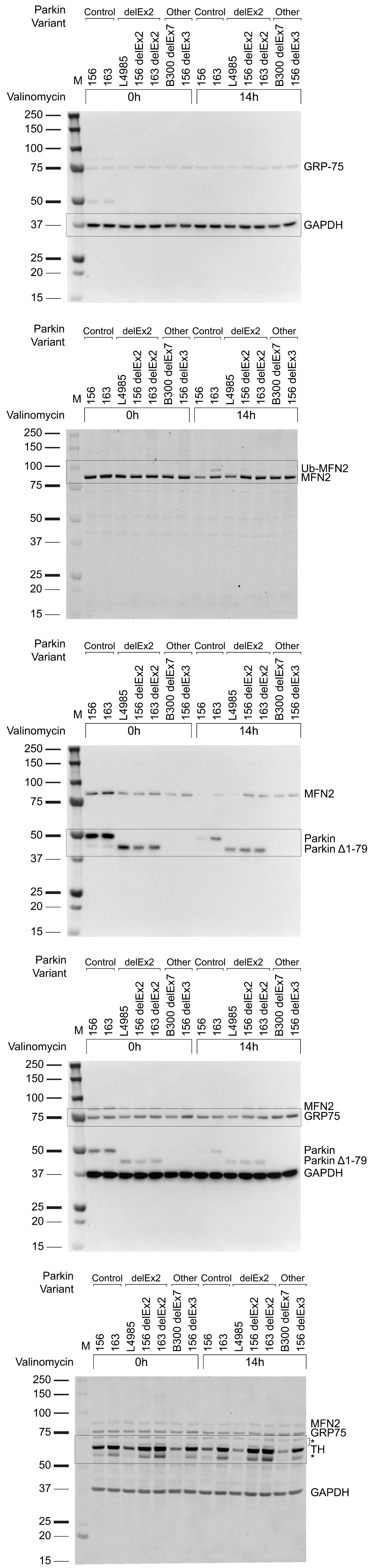

Complete western blots. Full blots after 6 h (**J**) and 14 h (**K**) of Valinomycin treatment in iDNs as shown in main figure 5. Blots are shown for each cropped signal, marked by boxes.

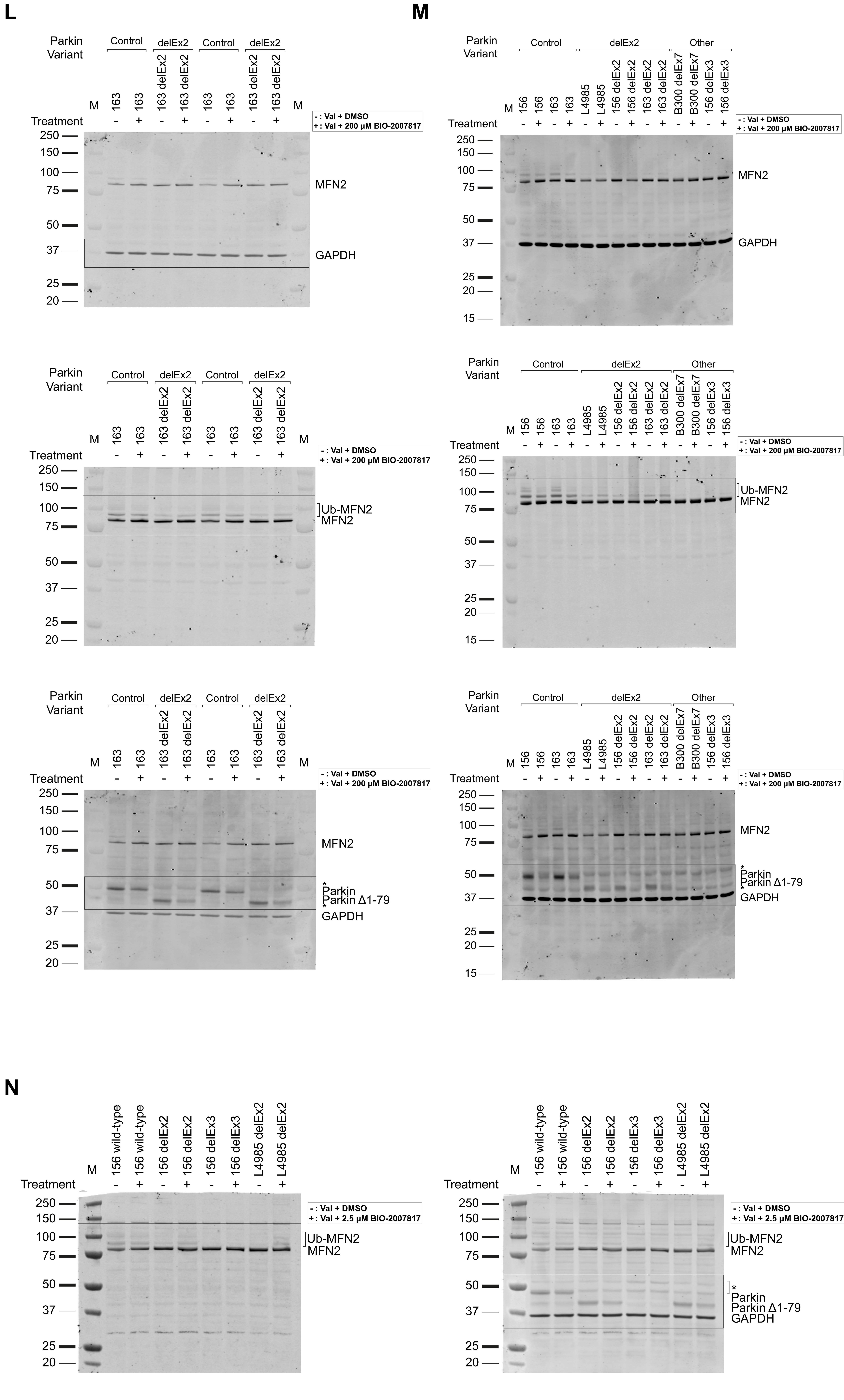

Complete western blots. Full western blots of BIO-2007817 experiments in iDNs and smNPCs as seen in main figure 6A left (**L**) and right (**M**), as well as 6H (**N**). Blots are shown for each cropped signal, marked by boxes.
